# Exercise blood pressure relative to fitness and cardiovascular outcomes: the EXERTION study

**DOI:** 10.1093/eurheartj/ehaf1082

**Published:** 2026-01-13

**Authors:** Martin G Schultz, Petr Otahal, Philip Roberts-Thomson, Tony Stanton, Christian Hamilton-Craig, Sudhir Wahi, Andre La Gerche, James L Hare, Joseph Selvanayagam, Andrew Maiorana, Alison J Venn, Thomas H Marwick, James E Sharman

**Affiliations:** Menzies Institute for Medical Research, University of Tasmania, Medical Science Precinct, 17 Liverpool Street, Hobart, Tasmania 7000, Australia; Menzies Institute for Medical Research, University of Tasmania, Medical Science Precinct, 17 Liverpool Street, Hobart, Tasmania 7000, Australia; Department of Cardiology, Royal Hobart Hospital, Hobart, Australia; Cardiology, Sunshine Coast University Hospital, Birtinya, Australia; University of Queensland, Brisbane, Australia; Department of Cardiology, Princess Alexandra Hospital, Brisbane, Australia; Baker Heart and Diabetes Institute, Melbourne, Australia; Baker Heart and Diabetes Institute, Melbourne, Australia; Department of Cardiology, The Alfred Hospital, Melbourne, Australia; Cardiac Imaging Research, Flinders University, Adelaide, Australia; Department of Cardiovascular Medicine, Flinders Medical Centre, Adelaide, Australia; Curtin School of Allied Health, Curtin University, Perth, Australia; Allied Health Department, Fiona Stanley Hospital, Perth, Australia; Menzies Institute for Medical Research, University of Tasmania, Medical Science Precinct, 17 Liverpool Street, Hobart, Tasmania 7000, Australia; Menzies Institute for Medical Research, University of Tasmania, Medical Science Precinct, 17 Liverpool Street, Hobart, Tasmania 7000, Australia; Baker Heart and Diabetes Institute, Melbourne, Australia; Menzies Institute for Medical Research, University of Tasmania, Medical Science Precinct, 17 Liverpool Street, Hobart, Tasmania 7000, Australia

**Keywords:** Blood pressure determination, Diagnostic techniques, Cardiovascular, Exercise tests, Mortality determinant

## Abstract

**Background and Aims:**

A hypertensive response to exercise is independently associated with cardiovascular disease (CVD), but clinical interpretation may be confounded by aerobic capacity (fitness). The aim of this study was to determine the relationship between exercise blood pressure (BP) relative to fitness and CVD events.

**Methods:**

Clinical exercise test records were analysed from 12 743 people (aged 53 ± 13 years, 60% male) who completed a standard exercise stress test (Bruce treadmill protocol, stages 1–4) at six Australian hospitals. Records were linked to administrative datasets (hospital and emergency admissions, death register) to define clinical characteristics and the primary outcome of fatal/non-fatal CVD events. Exercise systolic BP relative to fitness was calculated from the quotient of systolic BP and peak METs (SBP/MET_Peak_). Competing risks regression was undertaken to compare events across quartiles, at the 90th percentile, and at various thresholds of SBP/MET_Peak_.

**Results:**

Over a median follow-up of 51 months (interquartile range: 32–75 months), 1349 events occurred. Exercise systolic BP without consideration of fitness was not associated with cardiovascular events (*P* > .05). In models adjusted for age, sex, and pre-exercise systolic BP, there was a stepwise increase in cardiovascular events across SBP/MET_Peak_ quartiles at stages 1–3 and peak (fourth quarter hazard ratios [HR]: stage 1 HR 2.54, 95% confidence interval [CI] 2.08–3.12; stage 2 HR 2.05, 95% CI 1.64–2.57; stage 3 HR 1.60, 95% CI 1.22–2.10; peak HR 2.43, 95% CI 1.99–2.98). SBP/MET_Peak_ ≥90th percentile was associated with a 55–94% increased risk of cardiovascular events vs < 90th percentile (stages 1–3 and peak, *P* < .001). Thresholds from 15 to 24 mmHg/MET_Peak_ were associated with cardiovascular events in both males and females (*P* < .001, stages 1–3 and peak). Results persisted in those without CVD, normal pre-exercise BP, and in those on BP-lowering medication.

**Conclusions:**

Exercise systolic BP relative to fitness is associated with increased risk for cardiovascular events and could provide a clinically actionable marker to prompt targeted intervention to lower hypertension-related cardiovascular risk.

## Structured Graphical Abstract

**Structured Graphical Abstract:**
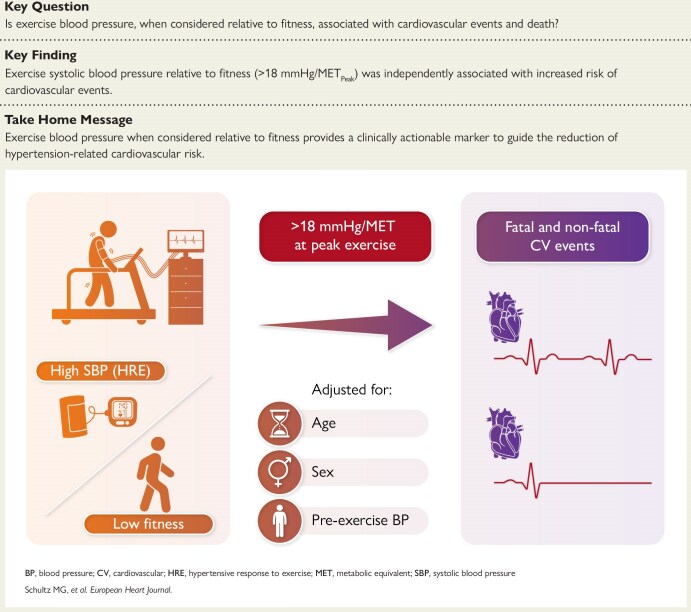

**See the editorial comment for this article ‘Hypertensive response to exercise and the theory of relativity', by H. Hanssen, https://doi.org/10.1093/eurheartj/ehag064.**

## Introduction

Clinical exercise stress testing is a common test performed in cardiology practice worldwide. While exercise testing is generally indicated to elicit the signs of exertional ischaemia, research over many years has also revealed associations between elevated systolic blood pressure (BP) recorded during exercise (termed a hypertensive response to exercise, HRE) and increased risk for cardiovascular disease (CVD) events and mortality independent from resting BP.^1–4^ The increased risk related to an HRE is likely attributable to uncontrolled or undetected high BP, its associated CVD risk factors, or the development of essential hypertension.^5–10^ However, not all studies have shown a consistent relationship between an HRE and adverse outcomes.^11–14^ The reasons accounting for these inconsistencies are not entirely understood, but may explain why clinical care pathways aimed at reducing CVD risk are generally not initiated in response to an individual having an HRE during clinical exercise stress testing.

It has recently been proposed that for appropriate clinical interpretation of an HRE, exercise BP should be considered relative to individual fitness level.^15^ Indeed, in people with high fitness, systolic BP does not tend to increase substantially during lower level incremental exercise,^16^ and an HRE is more likely to occur at higher exercise workloads (e.g. stage 3+ of a Bruce protocol). This is a normal physiological BP response, rather than a pathological sign of uncontrolled high BP associated with increased CVD risk that may be expected in people with lower levels of fitness. However, this conjecture has never been empirically tested. The aim of this study was, therefore, to determine the relationship between exercise BP considered relative to fitness and CVD outcomes (fatal and non-fatal CVD events) in a large clinical population of patients referred for standard hospital-based exercise stress testing. It was hypothesized that high exercise systolic BP (an HRE) relative to a low-level of fitness would be associated with increased CVD risk, thus potentially creating a clinically actionable CV risk marker.

## Methods

### Data source and inclusion

Analysis was performed using data from the EXERcise stress Test collaboratION (EXERTION) study. This is a national collaboration across six hospital sites (five public and one private) within Australia in which exercise stress testing data has been extracted, pooled, and linked to administrative health datasets to define clinical history and ascertain clinical outcomes. Exercise stress test data were extracted from three Queensland hospitals (date range 02/2002–02/2018), two Victorian hospitals (date range 01/2011–12/2019), and one Tasmanian hospital (date range 01/2011–12/2015). Individual patient exercise test data were linked by local (state based) data-linkage services with public (and private in Victoria) hospital admitted patient records (Queensland year range 2000–18, Tasmanian year range 2007–15, Victorian year range 2008–2018), public hospital emergency presentation records (Queensland year range 2009–18, Tasmanian year range 2000–15, Victorian year range 2008–2019), and the cause of death unit record file (Queensland year range 2009–18, Tasmanian year range 2000–15, Victorian year range 2005–2018). Ethical approval was gained from the University of Tasmania (H0015271), Metro South (HREC/16/QPAH/511), and Alfred (45 926) Human Research Ethics Committees, with all data extracted and linked under an approved waiver of consent.

**Figure 1:**
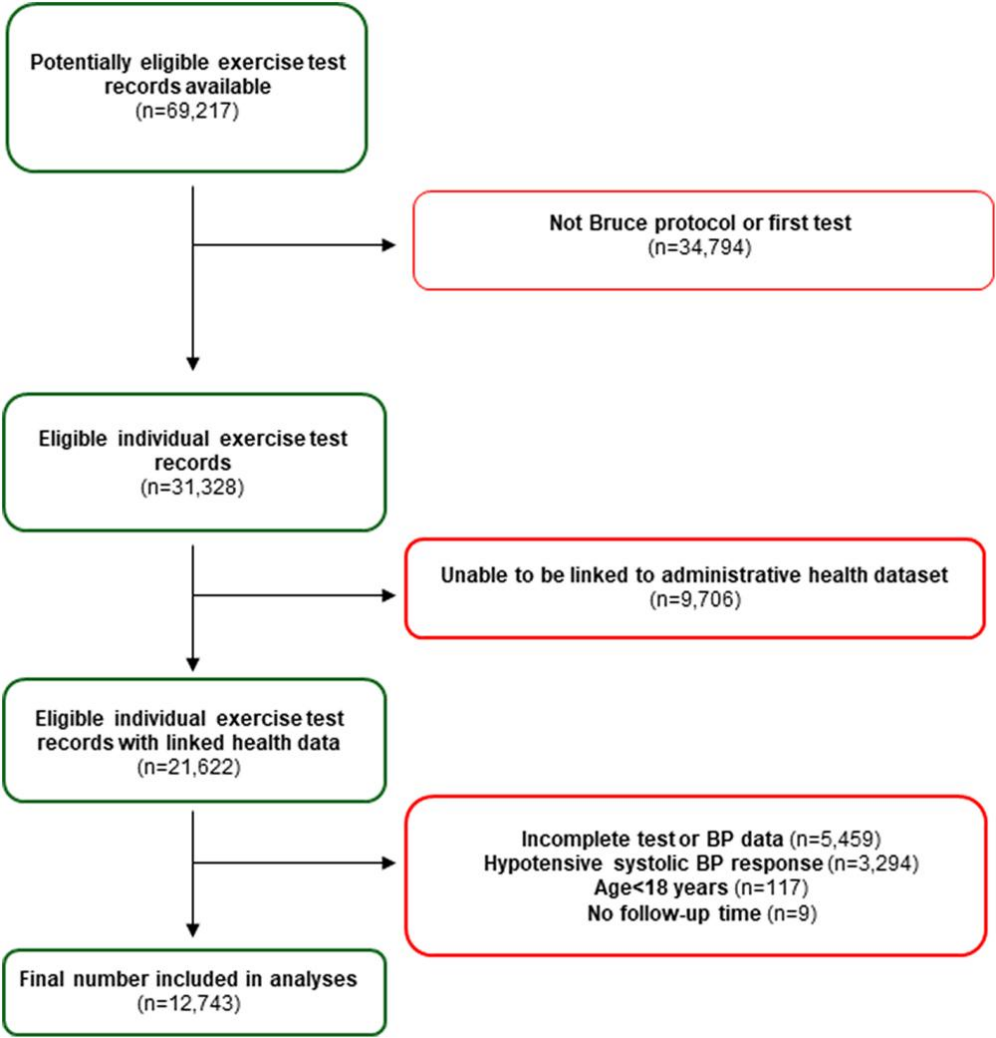
Study design and participant flow

*Figure 1* outlines the flow of participants for this analysis. There were 69 217 participants with potentially eligible exercise test data. Excluding tests that did not follow a standard Bruce treadmill protocol, were not the first exercise test, or in which a linkage to hospital admission or emergency presentation data was not possible, left a total of *n* = 21 622 eligible exercise tests. Those with incomplete exercise testing or BP data, a hypotensive response to exercise (defined as ≥10 mm Hg drop in systolic BP with increasing test stage), people <18 years of age, and people in whom there was no follow-up time (linked datasets not available following the exercise test) were further excluded, leaving a final *n* = 12 743 for analyses.

### Exercise testing and blood pressure

All exercise tests were standard, clinically indicated (e.g. for the assessment of symptoms related to ischaemia and/or arrythmia) exercise stress ECG tests undertaken with the Bruce treadmill protocol and following exercise testing guideline recommendations (American Heart Association and/or American College of Sports Medicine.^17,18^). The Bruce treadmill protocol involves walking on a treadmill at preset 3-min stages, whereby both speed and grade (treadmill incline) increase with each stage (stage 1 speed 2.7 km/h, incline 10%; stage 2 speed 4 km/h, incline 12%; stage 3 speed 5.5 km/h, incline 14%; stage 1 speed 6.8 km/h, incline 16%). As per guidelines, beta blocker medication was withheld on the day of testing.

All BP measurements were made according to clinical guideline recommendations. BP data from exercise stages 1, 2, 3, and 4 were included, as well as a maximum or ‘peak’ value which represented the highest BP value attained during testing, or the value of BP recorded immediately on test cessation. BP measures were included if there was at least one measure recorded at any of the four stages. This included a manual or automated auscultatory BP measurement at rest in either the supine, seated, or standing posture (pre-exercise BP), and during the last minute of each exercise stage. Analysis was focused on the systolic BP response to exercise testing because large changes can occur with exercise and it is the primary BP variable associated with clinical outcomes, whereas diastolic BP changes little with increasing exercise intensity.^19^ Exercise systolic BP relative to fitness at each exercise stage was calculated from the quotient of systolic BP and peak metabolic equivalents (METs) and expressed as mmHg/MET_Peak_. Peak METs was defined as the highest MET value reached for the exercise test according to Eq. 1, where the *speed* and *grade* are the treadmill data achieved at peak exercise for each individual. This is routinely calculated and provided as an output of the exercise stress testing software. A 1 One MET can be approximated as equal to 3.5 mL/kg/min^−1^, and for reference we have provided some data represented as systolic BP/mL/kg/min^−1^.


(1) METs=[(speed×0.1)+(grade/100×speed)+3.5]/3.5

### CVD outcomes

The primary outcome was a composite variable of fatal and non-fatal cardiovascular events (whichever occurred first after the exercise test). Non-fatal cardiovascular events were quantified from ICD-10 diagnosis codes within admitted patient or emergency presentation records following the date of first identified Bruce protocol exercise stress test for each individual. These included codes for acute myocardial infarction (I21, I22), atrial fibrillation (I48), heart failure (I50), and multiple forms of stroke (I60, I61, I63, I64). Fatal cardiovascular events (death) were ascertained at the state level from linked Cause of Death Unit Record File (COD-URF) datasets and judged to be CVD in origin if any of the aforementioned ICD-10 codes were listed as the primary cause of death. The competing risk outcome was all-cause mortality (death not of the above listed ICD-10 codes). Sensitivity analyses were also conducted with myocardial infarction and atrial fibrillation as individual outcome variables.

### CVD history and risk factors

Health history data were ascertained from linked administrative health datasets or the limited information provided with the exercise stress tests. A history of CVD was defined using ICD-10 codes [angina (I20), acute myocardial infarction (I21, I22), ischaemic heart disease (I25), cardiomyopathy (I42), atrial fibrillation (I48), heart failure (I50), cerebral infarction/stroke (I60, I61, I62, I63), and chronic kidney disease (N18)] appearing in the clinical history of admitted patient or emergency presentation reports prior to the first Bruce protocol exercise stress test. CVD risk factors prior to stress test [hypertension (I10), dyslipidemia (E78), and obesity (E66)] were recorded via the same process. The Duke treadmill score was calculated as exercise phase time (in minutes) – [(5* ST deviation (in mm)] – 4*angina index and grouped into risk categories (>+5 = low risk, −10 to +4 = intermediate risk, <−10 = high risk).

### Statistical analyses

Fine–Gray competing risks regression was used to model the risk of composite fatal and non-fatal CVD events across various levels of exercise systolic BP and exercise systolic BP relative to fitness. All-cause mortality (death from non-CVD cause) was considered a competing event. Subjects were considered at risk following the date of their first Bruce protocol stress test until the first failure to the composite endpoint. Missing data points were excluded from analyses at the individual variable level, rather than excluding the entire participant record. As per the approach taken in previous investigations, exercise systolic BP and exercise systolic BP relative to fitness were analysed across quartiles (with the first quarter as the reference category), at the 90th percentile to represent a traditional definition of an HRE (with <90th percentile as the reference category) and at various thresholds (with values below the threshold as the reference category). Models were adjusted for age, sex, pre-exercise systolic BP, and fitness (peak METs). The final models were tested to determine whether they satisfied the assumption of proportionality, via Martingale residuals.^20^ To demonstrate the individual predictive (diagnostic) value of systolic BP, peak METs, and SBP/MET_Peak_, a preliminary analysis to derive a C-statistic from an area under the curve (AUC) analysis (univariable) was reported with a predicted 50-month survival period. Sensitivity (subgroup) analyses were performed limiting the sample to those without a history of CVD, those with a pre-exercise BP of <130/80 mmHg, and adjusting for BP lowering medications in those that had available data on medication use at the time of the exercise stress test. The dataset was also dichotomized by the median exercise stress test date of 31 October 2013 to allow a results comparison between earlier and later performed exercise stress tests. The distributions of all variables were assessed visually via histograms and Q–Q distribution plots, with all distributions considered appropriate for the intended analysis. Analyses were conducted using R, version 4.1.2, using the cmprsk package for competing risk regression, with an alpha level set at .05.

## Results

### Participant characteristics

*Table 1* presents the demographic and clinical characteristics of participants. The sample was middle-to-older aged with a greater proportion of males. A CVD history was common (reflective of an exercise stress test referral population) with high prevalence of angina, previous myocardial infarction, and ischaemic heart disease. Almost one in five people had a documented history of hypertension. According to Duke treadmill score risk categorization, the cohort was most commonly at an intermediate risk level. Basic demographic and exercise tests data for those without linked data is also presented within Supplementary data online, *Table S1*.

**Table 1:** Demographic and clinical characteristics of study participants

*n*	12,743
Age (years)	52.8 (13)
Female sex, *n* (%)	5109 (40.1)
Fitness (MET_Peak_)	11.1 (3.4)
Fitness (VO_2Peak_ mm.kg.min^−1^)	38.85 (11.9)
Pre-exercise (at rest)	
Heart rate (bpm)	80 (14)
Systolic BP (mmHg)	127 (18)
Diastolic BP (mmHg)	76 (11)
Peak-exercise	
Heart rate (bpm)	162 (23)
Systolic BP (mmHg)	179 (25)
Diastolic BP (mmHg)	79 (13)
History of hypertension, *n* (%)	2346 (18.4)
History of dyslipidaemia, *n* (%)	1132 (8.9)
History of Type 2 diabetes	1086 (8.5)
History of obesity, *n* (%)	508 (4.0)
History of CVD, *n* (%)	7101 (55.8)
Angina, *n* (%)	5779 (45.4)
Acute myocardial infarction, *n* (%)	1331 (10.5)
Ischaemic heart disease, *n* (%)	2080 (16.3)
Cardiomyopathy, *n* (%)	151 (1.2)
Atrial fibrillation, *n* (%)	7.2 (5.5)
Heart failure, *n* (%)	183 (1.4)
Stroke, *n* (%)	132 (1.0)
Chronic kidney disease, *n* (%)	181 (1.4)
Duke Treadmill Score^a^	
Low risk (<−10), *n* (%)	1056 (15.0)
Intermediate risk (−10 to 9), *n* (%)	4992 (70.9)
High risk (10+), *n* (%)	997 (14.1)

Data are mean and standard deviation or *n* (percentage).

METs, metabolic equivalents; BP, blood pressure; CVD, cardiovascular disease.

### Outcomes

Over a median 51 (interquartile range 32–75) month study follow-up period, there was a total of 1349 combined fatal and non-fatal CVD events (ICD-10 coded event types listed in Supplementary data online, *Table S2*), with 193 competing risk events (deaths from non-CVD cause).

### Exercise systolic BP, fitness, fatal, and non-fatal CVD events

**Figure 2:**
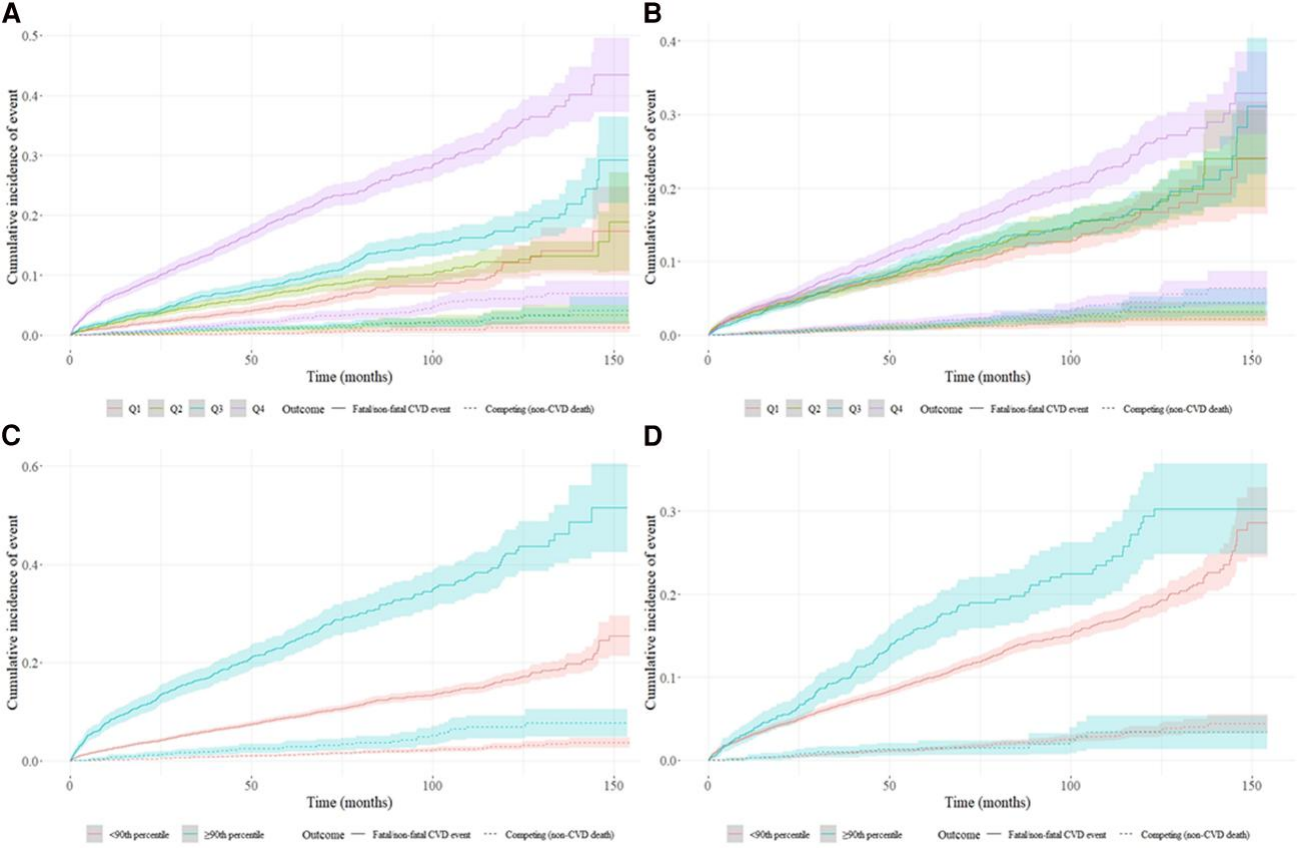
Cumulative incidence plots showing fatal/non-fatal CVD event incidence over time based on SBP/MET_Peak_ and competing risk (all-cause death); panel *A*) across quartiles of SBP/MET_Peak_, panel *B*) quartiles of systolic BP, panel *C*) at the 90th percentile of SBP/MET_Peak_, panel *D*) at the 90th percentile of systolic BP at stage 1 of a Bruce treadmill exercise test. The greatest incidence of fatal/non-fatal CVD events occur in the fourth quarter of SBP/MET_Peak_ (panel A) and ≥ 90^th^ percentile SBP/MET_Peak_ (panel C). See supplementary data online, *Tables S6*–9 for number of patients at risk

Fitness was inversely related with risk of fatal and non-fatal CVD events, with each additional 1 MET of peak workload achieved associated with a 13.9% reduced rate of the composite outcome when adjusted for age and sex (*P* < .001). The C-statistic for peak METs was 0.685. There was no change in relative risk of fatal and non-fatal CVD events across quartiles, or >90th percentile of exercise SBP evident at any exercise test stage (see Supplementary data online, *Table S3*, data at stage 1 presented in *Figure 2B* and *D*). This was consistent amongst both males and females (data at stage 1 presented in Supplementary data online, *Figures S1B* and *S1D* for females, 2B and 2D for males) and within subsamples of those without raised pre-exercise BP, a CVD history, or when adjusting for BP lowering medication (see Supplementary data online, *Table S3*). The C-statistic for systolic BP at stage 1 was 0.537. The mathematical assumptions related to representing SBP and peak METs as a ratio were also assessed (see Supplementary data online, *Figure S3*), where the relationship between stage 1 SBP and peak METs was such that as fitness (peak METs) increased, the exercise SBP at stage 1 of the Bruce protocol reduced.

### Exercise SBP/MET_Peak_, fatal, and non-fatal CVD events

Compared with the lowest quarter of exercise SBP/MET_Peak_ (reference), relative risk of fatal and non-fatal CVD events increased stepwise across quartiles at exercise test stages 1–3 and peak, with the strongest associations found in the fourth quarter across each of those stages (*Table 2*). An example of this risk discrimination at exercise stage 1 is depicted in *Figure 2A*, where the highest event rate occurs in the fourth quarter of SBP/MET_Peak_. These results persisted with adjustment for age and sex (*Table 2*, model 2), with additional adjustment for pre-exercise systolic BP (*Table 2*, model 3), when the sample was restricted to those without a history of CVD (*Table 2*, model 4), in those with pre-exercise BP <130/80 mmHg (*Table 2*, model 5), and when adjusting for potential BP lowering medications in the subsample with medication information available (*Table 2*, model 6). Results were relatively consistent amongst both males (see Supplementary data online, *Table S4*, Supplementary data online, *Figure S1A*) and females (see Supplementary data online, *Table S5*, Supplementary data online, *Figure S2A*).

**Table 2:** Relative risk of fatal/nonfatal CVD events across quartiles and the 90th percentile of SBP/MET_Peak_ at each exercise test stage

SBP/MET_Peak_	Model 1	Model 2	Model 3	Model 4	Model 5	Model 6
	HR (95% CI)	HR (95% CI)	HR (95% CI)	HR (95% CI)	HR (95% CI)	HR (95% CI)
**Stage 1 (*n* = 12 686)**						
1st Quarter	1.00 (Ref)	1.00 (Ref)	1.00 (Ref)	1.00 (Ref)	1.00 (Ref)	1.00 (Ref)
2nd Quarter	1.34 (1.10–1.64)	1.09 (0.89–1.34)	1.14 (0.93–1.39)	0.90 (0.64–1.27)	1.07 (0.84–1.36)	1.01 (0.66–1.54)
3rd Quarter	1.83 (1.52–2.20)	1.31 (1.07–1.59)	1.41 (1.15– 1.72)	1.17 (0.85–1.62)	1.45 (1.14–1.83)	1.04 (0.69–1.58)
4th Quarter	4.00 (3.38–4.73)	2.27 (1.87–2.76)	2.54 (2.08– 3.12)	1.77 (1.29–2.44)	2.46 (1.94–3.10)	1.60 (1.05–2.44)
<90th Percentile	1.00 (Ref)	1.00 (Ref)	1.00 (Ref)	1.00 (Ref)	1.00 (Ref)	1.00 (Ref)
≥90th Percentile	3.01 (2.65–3.42)	1.85 (1.61–2.14)	1.94 (1.67–2.25)	1.93 (1.54–2.43)	2.21 (1.82–2.69)	1.67 (1.18–2.37)
**Stage 2 (*n* = 11 349)**						
1st Quarter	1.00 (Ref)	1.00 (Ref)	1.00 (Ref)	1.00 (Ref)	1.00 (Ref)	1.00 (Ref)
2nd Quarter	1.38 (1.12–1.71)	1.12 (0.90–1.39)	1.16 (0.93–1.44)	0.87 (0.60–1.25)	1.20 (0.93–1.56)	1.00 (0.64–1.56)
3rd Quarter	1.77 (1.45–2.17)	1.30 (1.05–1.61)	1.38 (1.11–1.71)	1.16 (0.82–1.64)	1.37 (1.05–1.78)	1.03 (0.67–1.57)
4th Quarter	3.12 (2.58–3.77)	1.87 (1.52–2.32)	2.05 (1.64–2.57)	1.46 (1.03–2.09)	2.06 (1.58–2.68)	1.40 (0.91–2.15)
<90th Percentile	1.00 (Ref)	1.00 (Ref)	1.00 (Ref)	1.00 (Ref)	1.00 (Ref)	1.00 (Ref)
≥90th Percentile	2.35 (2.00–2.75)	1.55 (1.31–1.84)	1.60 (1.35–1.91)	1.42 (1.05–1.91)	1.71(1.33–2.20)	1.41 (1.05–1.89)
**Stage 3 (*n* = 9175)**						
1st Quarter	1.00 (Ref)	1.00 (Ref)	1.00 (Ref)	1.00 (Ref)	1.00 (Ref)	1.00 (Ref)
2nd Quarter	1.39 (1.08–1.78)	1.15 (0.89–1.48)	1.17 (0.90–1.51)	1.22 (0.80–1.86)	1.23 (0.91–1.67)	0.99 (0.59–1.64)
3rd Quarter	1.76 (1.38–2.23)	1.32 (1.03–1.70)	1.37 (1.06–1.77)	1.37 (0.89–2.11)	1.39 (1.02–1.91)	0.86 (0.51–1.45)
4th Quarter	2.31 (1.83–2.91)	1.50 (1.16–1.95)	1.60 (1.22–2.10)	1.50 (0.96–2.33)	1.57 (1.12–2.19)	0.84 (0.50–1.41)
<90th Percentile	1.00 (Ref)	1.00 (Ref)	1.00 (Ref)	1.00 (Ref)	1.00 (Ref)	1.00 (Ref)
≥90th Percentile	2.07 (1.67–2.56)	1.50 (1.20–1.88)	1.55 (1.22–1.97)	1.46 (0.95–2.24)	1.63 (1.14–2.34)	1.26 (0.84–1.89)
**Stage 4 (*n* = 5216)**						
1st Quarter	1.00 (Ref)	1.00 (Ref)	1.00 (Ref)	1.00 (Ref)	1.00 (Ref)	1.00 (Ref)
2nd Quarter	1.11 (0.78–1.60)	0.92 (0.64–1.33)	0.94 (0.65–1.36)	1.06 (0.57–1.95)	0.97 (0.63–1.47)	0.61 (0.30–1.24)
3rd Quarter	1.25 (0.87–1.78)	0.86 (0.60–1.24)	0.89 (0.62–1.29)	1.09 (0.61–1.97)	0.92 (0.59–1.43)	0.56 (0.28–1.11)
4th Quarter	1.60 (1.13–2.26)	0.92 (0.64–1.32)	0.97 (0.67–1.41)	1.45 (0.79–2.64)	0.85 (0.52–1.38)	0.58 (0.28–1.18)
<90th Percentile	1.00 (Ref)	1.00 (Ref)	1.00 (Ref)	1.00 (Ref)	1.00 (Ref)	1.00 (Ref)
≥90th Percentile	1.91 (1.36–2.69)	1.27 (0.89–1.80)	1.33 (0.92–1.93)	1.71 (0.93–3.17)	1.25 (0.73–2.15)	0.78 (0.39–1.56)
**Peak (*n* = 12 717)**						
1st Quarter	1.00 (Ref)	1.00 (Ref)	1.00 (Ref)	1.00 (Ref)	1.00 (Ref)	1.00 (Ref)
2nd Quarter	1.42 (1.16–1.73)	1.11 (0.91–1.36	1.15 (0.94–1.40)	1.14 (0.81–1.60	1.04 (0.82–1.32)	0.82 (0.54–1.26)
3rd Quarter	2.13 (1.77–2.57)	1.47 (1.21–1.79)	1.56 (1.28–1.90)	1.56 (1.13–2.16)	1.49 (1.18–1.88)	0.98 (0.65–1.47)
4th Quarter	4.07 (3.42–4.83)	2.22 (1.83–2.69)	2.43 (1.99–2.98)	2.02 (1.45–2.80)	2.29 (1.81–2.89)	1.36 (0.89–2.06)
<90th Percentile	1.00 (Ref)	1.00 (Ref)	1.00 (Ref)	1.00 (Ref)	1.00 (Ref)	1.00 (Ref)
≥90th Percentile	2.74 (2.41–3.12)	1.64 (1.42–1.89)	1.69 (1.46–1.97)	1.51 (1.19–1.92)	2.01 (1.65–2.45)	1.23 (0.086–1.75)

Model 1 is univariable. Model 2 is adjusted for age and sex. Model 3 is adjusted for age, sex, and pre-exercise systolic BP. Model 4 is adjusted for age and sex and limited to those with no CVD history (*n* = 2147–5627 across stages). Model 5 is adjusted for age and sex and limited to those with a pre-exercise systolic BP <140/90 mmHg (*n* = 3414–7804 across stages). Model 6 is adjusted for age, sex, and potential BP lowering medication (yes/no), limited to those with current medications reported (*n* = 1107–2492 across stages).

SBP/MET_Peak_ ≥90th percentile was associated with an increased fatal and non-fatal CVD event rate vs <90th percentile across exercise test stages 1–3 and peak (*Table 2*). Results persisted across models 1–6 with adjustment for age, sex, pre-exercise systolic BP, and within the subgroups without a history of CVD (stages 1–2 and peak), pre-exercise BP <130/80 mmHg (stages 1–3 and peak), and with adjustment for BP lowering medication (stages 1–2). These findings were consistent irrespective of sex (see Supplementary data online, *Tables S4* and *S5*, Supplementary data online, *Figures S1C* and *S2C*).

A continuous sensitivity analysis was also performed with SBP/MET_Peak_ at stage 1, with results indicating only a modest per-unit increased risk for fatal and non-fatal CV events after adjustment for age, sex, and resting BP (HR 1.02, 95% CI 1.01–1.02). Despite this, the area under the curve (AUC) for the continuous variable SBP/MET_Peak_ at exercise test stage 1 in the prediction of fatal and non-fatal CV events was [67.1% (95% CI 65.2–69.0%)]. In a sub-analysis of 4138 participants, a recovery SBP/MET_Peak_ >90th percentile, adjusted for age, sex, and pre-exercise SBP was associated with increased risk for fatal and non-fatal CVD events (HR 2.33, 95% CI 1.65–3.31).

### Thresholds of exercise SBP/MET_Peak_

**Figure 3:**
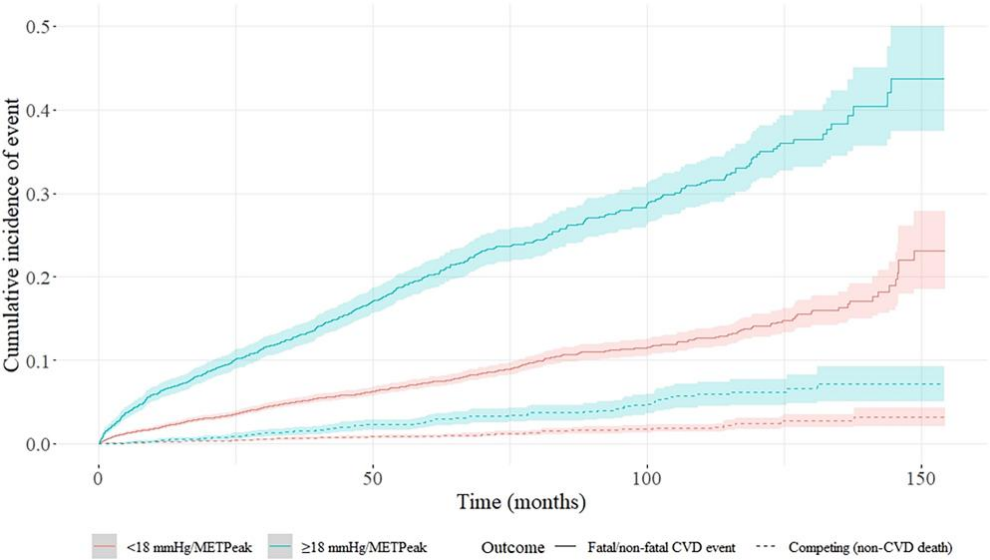
Cumulative incidence plots showing fatal/non-fatal CVD event incidence over time based on a threshold of 18 mmHg/MET_Peak_ at stage 1 of a Bruce treadmill exercise test. SBP/MET_Peak_ ≥ 18 mmHg/MET_Peak_ (blue line) shows strong and clear delineation of CVD risk compared with <18 mmHg/MET_Peak_ (pink line). See supplementary data online, *Tables S10* for number of patients at risk

SBP/MET_Peak_ at the 75th and 90th percentile was higher in females vs males across each exercise test stage (*Table 3A*). Relative risk of fatal and non-fatal CVD events at various thresholds of SBP/MET_Peak_ across each exercise test stage is presented in *Table 3B*. A range of SBP/MET_Peak_ thresholds from 15 to 24 mmHg/MET_Peak_ (adjusted for age, sex, and pre-exercise systolic BP) show increased risk for fatal and non-fatal CVD events across exercise test stages 1–3 and peak. Risk of fatal and non-fatal CVD events were particularly evident during stage 1, where there was near a doubling of event risk (*Table 3B*), with this clear risk discrimination highlighted at a threshold of 18 mmHg/MET_Peak_ in *Figure 3*. All results were consistent amongst males and females (stages 1–3 and peak) and persisted within the subgroup analyses (see Supplementary data online, *Table S6*), in those without a history of CVD (stages 1–2 and peak), pre-exercise BP <130/80 mmHg (stages 1–2 and peak) and when adjusting for BP lowering medication (stage 1).

**Table 3:** The 75th and 90th percentiles of SBP/MET_Peak_ and SBP/VO_2Peak_ for each stage and the relative risk of fatal/nonfatal CVD events event or death at various cut-points of across each exercise test stage

A.	mmHg/MET_Peak_		mmHg/mL.kg.min^−1^		B.	≥15 mmHg/MET_Peak_ ≥4.29 mmHg/mL.kg.min^−1^	≥18 mmHg/MET_Peak_ ≥5.14 mmHg/mL.kg.min^−1^	≥20 mmHg/MET_Peak_ ≥5.71 mmHg/mL.kg.min^−1^	≥24 mmHg/MET_Peak_ ≥6.86 mmHg/mL.kg.min^−1^
All^a^	Percentile		Percentile						
	75th	90th	75th	90th		HR (95% CI)	HR (95% CI)	HR (95% CI)	HR (95% CI)
Stage 1	17.60	23.91	5.03	6.83		1.94 (1.70–2.20)	1.93 (1.70– 2.20)	1.97 (1.72– 2.25)	1.93 (1.66–2.25)
Stage 2	18.00	22.54	5.14	6.44		1.52 (1.32–1.76)	1.65 (1.43– 1.91)	1.60 (1.37– 1.86)	1.67 (1.38–2.03)
Stage 3	17.05	19.72	4.87	5.63		1.20 (1.01–1.43)	1.39 (1.13– 1.70)	1.62 (1.27– 2.07)	2.28 (1.58–3.28)
Stage 4	15.00	17.24	4.29	4.93		1.04 (0.79–1.39)	1.45 (0.97– 2.19)	1.49 (0.85– 2.62)	1.05 (0.36–3.04)
Peak	20.64	26.82	5.90	7.66		1.44 (1.25–1.66)	1.87 (1.65– 2.13)	1.82 (1.61– 2.07)	1.82 (1.58–2.08)
**Female**									
Stage 1	19.09	25.71	5.45	7.35		2.14 (1.65–2.77)	2.32 (1.82–2.95)	2.13 (1.66–2.73)	2.01 (1.53–2.63)
Stage 2	18.95	23.57	5.41	6.73		1.59 (1.18–2.14)	1.82 (1.36–2.43)	1.98 (1.47–2.67)	1.53 (1.06–2.22)
Stage 3	17.63	20.24	5.04	5.78		1.16 (0.79–1.68)	1.05 (0.66–1.67)	1.62 (0.98–2.68)	2.05 (1.01–4.14)
Stage 4	15.32	17.45	4.38	4.99		0.71 (0.38–1.33)	1.59 (0.69–3.65)	2.44 (0.86–6.93)	1.66 (0.28–9.65)
Peak	22.00	28.44	6.29	8.13		1.53 (1.14–2.04)	1.91 (1.48–2.46)	2.00 (1.57–2.54)	1.87 (1.45–2.42)
**Male**									
Stage 1	16.70	22.40	4.77	6.40		1.87 (1.61–2.18)	1.79 (1.53–2.09)	1.89 (1.62– 2.22)	1.88 (1.57–2.26)
Stage 2	17.44	21.57	4.98	6.16		1.50 (1.27–1.77)	1.59 (1.35–1.88)	1.46 (1.22–1.76)	1.73 (1.38–2.17)
Stage 3	16.67	19.42	4.76	5.55		1.22 (1.00–1.47)	1.51 (1.20–1.90)	1.63 (1.23–2.15)	2.42 (1.58–3.71)
Stage 4	14.85	17.14	4.24	4.90		1.14 (0.84–1.57)	1.41 (0.88–2.26)	1.20 (0.60–2.42)	0.76 (0.18–3.19)
Peak	19.81	25.49	5.66	7.28		1.43 (1.22–1.68)	1.87 (1.61–2.16)	1.76 (1.51–2.04)	1.78 (1.51–2.10)

A. Data are SBP/MET_Peak_ (mmHg/MET_Peak_) and SBP/VO_2Peak_ (mmHg/mL.kg.min^−1^) at the 75th and 90th percentiles of each exercise test stage. B. Data are hazard ratios and 95% confidence intervals adjusted for age and pre-exercise SBP. Reference group is <each cut-point value.

^a^Models also adjusted for sex.

Further sensitivity analyses were undertaken with consideration of test date, age, and individual disease outcome. An SBP/MET_Peak_ ≥18 mmHg/MET_Peak_ at exercise test stage 1 was associated with increased risk of fatal and non-fatal CVD events (adjusted for age, sex, and pre-exercise systolic BP) within both the earlier (pre-median test date HR 1.87, 95% CI 1.55–2.11) and later exercise test groups (post-median test date HR 2.20, 95% CI 1.73–2.81). An SBP/MET_Peak_ ≥18 mmHg/MET_Peak_ at exercise test stage 1 was associated with increased risk of fatal and non-fatal CVD events (adjusted for age, sex, and pre-exercise systolic BP) to similar extent within both the younger (below median age HR 1.02, 95% CI 1.01–1.03) and older age groups (above median age HR 1.02, 95% CI 1.01–1.02). With adjustment for age, sex, and pre-exercise systolic BP, an SBP/MET_Peak_ ≥18 mmHg/MET_Peak_ at exercise test stage 1 was associated with increased risk of myocardial infarction (HR 1.91, 95% CI 1.52–2.40) and atrial fibrillation (HR 1.80, 95% CI 1.50–2.16) as individual outcome variables.

## Discussion

In this large clinical exercise stress testing population, an HRE considered relative to fitness (SBP/MET_Peak_) was found to be associated with increased risk of fatal and non-fatal CVD events. The increased risk associated with an HRE was not seen when considering exercise systolic BP without indexation to fitness (*Structured Graphical Abstract*). The combination of low fitness with high exercise systolic BP during early exercise test stages could provide a clinically actionable marker that prompts follow-up investigation to ascertain BP control and clinical care to lower CVD risk.

The results of this study are the first to describe exercise BP considered relative to fitness as a discriminator of CVD risk related to an HRE. As hypothesized, this risk discrimination was particularly evident during the earlier exercise test stages, where the deleterious effects of low fitness, combined with an HRE were associated with substantially increased CVD risk. Low fitness, assessed during clinical exercise stress testing, is one of the strongest and most important risk factors for CVD morbidity and mortality.^21,22^ To this end, the American Heart Association supports fitness assessment to be a ‘vital sign’ of CVD health and broadly encourages its measurement in general CVD risk screening.^23^ However, the measurement of fitness in and of itself rarely drives clinical decision-making or therapeutic pathways. Moreover, low fitness assessed in isolation is not a clinical indication to assess BP control (where much of the associated CVD risk from an HRE may reside). The key new finding of this study is that assessment of fitness together with exercise BP is of vital importance to gain an appropriate clinical understanding of an HRE.^15^

While a physiologically ‘normal’ exercise systolic BP response to graded exercise testing is in the order of 8 mmHg for every 1 MET increase in intensity,^16^ the rate of systolic BP increase is greater through early exercise testing stages in people with low fitness, with an HRE likely to occur after only a short duration of exercise, and at a low relative workload (e.g. stages 1–2 of a Bruce protocol). Conversely, with higher fitness, the rate of increase in systolic BP is lower, with a maximal BP response (a potential HRE) occurring after a greater duration of exercise and at a higher workload (e.g. stages 3+ of a Bruce protocol). Without knowledge of fitness, there is no way to determine if the HRE recorded is a benign physiological consequence of healthy exercise capacity,^24,25^ or a potential pathological sign of impaired cardiovascular function (uncontrolled high BP) requiring follow-up care.^10^ This study emphasizes the value of routinely determining fitness in METs from the exercise test software coupled with exercise BP for better CVD risk discrimination related to an HRE. This could not be achieved with METs or exercise BP as separate entities, and using an exercise systolic BP/fitness ratio overcomes methodological limitations associated with absolute systolic BP thresholds to define an HRE at peak/maximal intensity exercise,^16^ where the influence of cardiorespiratory fitness cannot be accounted for.

The measurement of BP during clinical exercise testing is a guideline mandated safety requirement, with abnormal responses forming a relative test-termination criteria.^18,26^ However, clinical decisions and pathways of care based around the measured exercise BP (including an HRE) are seldom implemented. Indeed, hypertension management guidelines cite various limitations to the clinical evaluation of exercise BP, including lack of standardized measurement methodologies, ill-defined ‘normal’ responses and a lack of consistent CVD risk discrimination.^27^ There has also been a traditional focus on absolute HRE thresholds based on BP measured at peak/maximal exercise,^10^ and more recently workload-indexed definitions that are dependent on the baseline systolic BP and peak workload achieved. All have shown varying association with CVD outcomes.^28–30^ Nonetheless, considering the BP response relative to fitness in this analysis showed SBP/MET_Peak_ to be associated with increased CVD risk across a wide range of values (see *Table 3*), with little difference in CVD risk discrimination beyond thresholds >75th percentile (corresponding to approximately 15 mmHg/MET_Peak_), and most notably at any exercise test stage. Furthermore, although females showed marginally higher values of SBP/MET_Peak_ the level of CVD risk was comparable to males at each threshold, again highlighting the importance of considering both BP and fitness.

Consensus on an HRE threshold associated with increased CVD risk has proved challenging. Much of the known CVD risk related to an HRE can be attributed to underlying or uncontrolled high BP and hypertension-related CVD risk.^10^ With approximately one-third of the adult population likely to have uncontrolled high BP,^31,32^ and assuming majority of such people may present with an HRE, there would be clinical merit in selecting an HRE threshold close to the 70th percentile, with the aim of capturing as many at-risk patients as possible. On the other hand, a higher threshold may hold greater sensitivity for detecting cases of uncontrolled high BP and elevated CVD risk but would trade-off the potential to identify all patients at increased CVD risk. Based on the results of the present study, a threshold of 18 mmHg/MET_Peak_ is conservatively proposed to denote increased risk related to uncontrolled high BP warranting follow-up care. This value approximates >75th percentile across exercise test stages 1–3 in the study population, while also clearly delineating increased CVD risk (particularly evident during stages 1 and 2) in both males and females (negating requirement to have sex-specific thresholds). Nonetheless, our data also indicates a relative stepwise increase in risk across quartiles of SBP/MET_Peak_. Before definitive recommendations can be made, further work is required to understand the diagnostic accuracy (sensitivity and specificity) of SBP/MET_Peak_ thresholds to appropriately classify those with uncontrolled hypertension (via out-of-office BP measures).

There are some limitations to the present study. METs were derived from speed and grade of the treadmill to provide an estimate of fitness. This has the advantage of being easily quantified from standardized clinical exercise testing protocols with minimal resources (such as the widely utilized Bruce treadmill protocol). While we provided estimated VO_2_ data (1 MET = 3.5 mL.kg.min^−1^) in our tables, future studies should explore the effect of indexing the exercise BP response to various fitness measures, as it remains unknown if objectively measured fitness (such as VO_2Peak_ derived from cardiopulmonary exercise testing) would produce different results. Furthermore, since fitness (MET_Peak_) was also associated with increased risk of fatal and non-fatal CVD events, it is possible that some of the risk related to SBP/MET_Peak_ is attributable to poor fitness. However, this is likely clinically inconsequential because fitness alone does not drive clinical decisions around BP management, yet intervention to improve fitness will lower BP and improve related CVD risk. ICD-10 diagnosis and procedure codes were used to characterize patient CVD risk factors and general health history prior to exercise testing as there was an absence of deeper clinical phenotyping from within the EXERTION dataset (e.g. measures of body composition, blood biochemistry). Administrative health datasets are also prone to under ascertainment of CV risk factors, and it is therefore possible that some factors not characterized may have influenced the results. Only a limited assessment of selection bias in relation to the presence/absence of linked health data was possible. Whilst there were largely no clinically meaningful differences in key exercise test variables in those who completed a Bruce treadmill protocol but in whom there was no linked data available (compared with the final included sample), the broader applicability of results to other testing protocols and populations remains to be assessed. Similarly, treatment and follow-up procedures from exercise stress testing may have differed across the timeline of the study, potentially contributing to time-varying confounding. However, we are unable to account for this within our analysis due to absence of this data. Detailed medication data (such as indication, duration of use, dosage, and compliance) was not available from within the full sample. However, antihypertensive medication does not necessarily curtail an HRE,^33,34^ and the subgroup analyses adjusting for potential BP lowering medications (see model 6, *Table 2*) did not significantly change results during early exercise test stages. We were also unable to assess the physiological process that may underlie an HRE and its associated CVD risk in this study, which may be of particular importance within high-risk individuals, in whom increased BP during exercise may be protective. Since BP measurements in recovery from exercise testing were not performed in a standardized manner (differing with respect to intensity, posture, and time), results should be viewed as preliminary only. Adjustments for multiple comparisons were not made, although statistical significance (*P* < .05) is not reported nor emphasized in the interpretation of results. Finally, we chose to represent the dependency of exercise SBP on fitness as a ratio variable (SBP/MET_Peak_). While this has the advantage of clinical simplicity, the mathematical assumptions of the ratio were not met because the relationship between the numerator (SBP) and denominator (MET_Peak_) was not a straight line through the origin (see Supplementary data online, *Figure S3*). Despite this, the observed relationship between exercise SBP and peak METs was not unexpected and highlights a practical inability to ‘de-couple’ the two variables. There may be other methods to account for the influence of fitness on exercise SBP which may produce different results, and further studies should aim to determine this.

## Conclusion/perspective

In most tertiary healthcare settings, the measurement of BP during clinical exercise testing is ubiquitous and fully integrated into testing protocols and systems. In this study, considering an HRE relative to fitness revealed strong associations with CVD events and death amongst a large population referred for standard clinical exercise stress testing via the widely completed Bruce treadmill protocol. Thus, coupling the prognostic power of fitness with the exercise BP response provides a potential clinical marker upon which to assess CVD risk related to uncontrolled high BP that cannot be determined from exercise BP and fitness alone.

## Supplementary Material

ehaf1082_Supplementary_Data

## Declarations

### Data Availability

Data related to this manuscript may be made available upon reasonable request to the corresponding author.

## References

[1] Schultz MG, Otahal P, Cleland VJ, Blizzard L, Marwick TH, Sharman JE. Exercise-induced hypertension, cardiovascular events, and mortality in patients undergoing exercise stress testing: a systematic review and meta-analysis. Am J Hypertens 2013;26:357–66. 10.1093/ajh/hps05323382486 23382486

[2] Filipovsky J, Ducimetiere P, Safar ME. Prognostic significance of exercise blood pressure and heart rate in middle-aged men. Hypertension 1992;20:333–9. 10.1161/01.HYP.20.3.333 1387630

[3] Laukkanen JA, Kurl S, Rauramaa R, Lakka TA, Venalainen JM, Salonen JT. Systolic blood pressure response to exercise testing is related to the risk of acute myocardial infarction in middle-aged men. Eur J Cardiovasc Prev Rehabil 2006;13:421–8. 10.1097/01.hjr.0000198915.83234.59 16926673

[4] Mariampillai JE, Engeseth K, Kjeldsen SE, Grundvold I, Liestøl K, Erikssen G, et al Exercise systolic blood pressure at moderate workload predicts cardiovascular disease and mortality through 35 years of follow-up in healthy, middle-aged men. Blood Press 2017;26:229–36. 10.1080/08037051.2017.1291276 28276720

[5] Moore MN, Climie RE, Otahal P, Schultz MG. Exercise blood pressure and cardiovascular disease risk: a systematic review and meta-analysis of cross-sectional studies. J Hypertens 2021;39:2395–402. 10.1097/hjh.0000000000002962 34738988

[6] Moore MN, Climie RE, Otahal P, Sharman JE, Schultz MG. Exercise blood pressure and cardiac structure: a systematic review and meta-analysis of cross-sectional studies. J Sci Med Sport 2021;24:925–30. 10.1016/j.jsams.2021.02.014 33707155

[7] Lim PO, Donnan PT, MacDonald TM. How well do office and exercise blood pressures predict sustained hypertension? A Dundee Step Test Study. J Hum Hypertens 2000;14:429–33. 10.1038/sj.jhh.1001041 10918547

[8] Manolio TA, Burke GL, Savage PJ, Sidney S, Gardin JM, Oberman A. Exercise blood pressure response and 5-year risk of elevated blood pressure in a cohort of young adults: the CARDIA study. Am J Hypertens 1994;7:234–41. doi: 0895-7061(94)90104-X [pii] 8003274 10.1093/ajh/7.3.234

[9] Schultz MG, Otahal P, Picone DS, Sharman JE. Clinical relevance of exaggerated exercise blood pressure. J Am Coll Cardiol 2015;66:1843–5. 10.1016/j.jacc.2015.08.015 26483112

[10] Schultz MG, La Gerche A, Sharman JE. Blood pressure response to exercise and cardiovascular disease. Curr Hypertens Rep 2017;19:89. 10.1007/s11906-017-0787-1 29046978

[11] Lauer MS, Pashkow FJ, Harvey SA, Marwick TH, Thomas JD. Angiographic and prognostic implications of an exaggerated exercise systolic blood pressure response and rest systolic blood pressure in adults undergoing evaluation for suspected coronary artery disease. J Am Coll Cardiol 1995;26:1630–6. 10.1016/0735-1097(95)00388-6 7594096

[12] Lauer MS, Levy D, Anderson KM, Plehn JF. Is there a relationship between exercise systolic blood pressure response and left ventricular mass? The framingham heart study. Ann Intern Med 1992;116:203–10. 10.7326/0003-4819-116-3-203 1530806

[13] Lewis GD, Gona P, Larson MG, Plehn JF, Benjamin EJ, Donnell O, et al Exercise blood pressure and the risk of incident cardiovascular disease (from the Framingham Heart Study). Am J Cardiol 2008;101:1614–20. 10.1016/j.amjcard.2008.01.046 18489939 PMC2953798

[14] Hedman K, Lindow T, Cauwenberghs N, Carlén A, Elmberg V, Brudin L, et al Peak exercise SBP and future risk of cardiovascular disease and mortality. J Hypertens 2022;40:300–9. 10.1097/hjh.0000000000003008 34475344 PMC8728754

[15] Schultz MG, La Gerche A, Sharman JE. Cardiorespiratory fitness, workload, and the blood pressure response to exercise testing. Exerc Sport Sci Rev 2022;50:25–30. 10.1249/JES.0000000000000276 34669623

[16] Currie KD, Floras JS, La Gerche A, Goodman JM. Exercise blood pressure guidelines: time to Re-evaluate what is normal and exaggerated? Sports Med 2018;48:1763–71. 10.1007/s40279-018-0900-x 29574665

[17] American College of Sports Medicine: Guidelines for Exercise Testing and Prescription. 9th edn. Lippincott: Williams & Wilkins, 2013.

[18] Fletcher GF, Ades PA, Kligfield P, Arena R, Balady GJ, Bittner VA, et al Exercise standards for testing and training: a scientific statement from the American Heart Association. Circulation 2013;128:873–934. 10.1161/CIR.0b013e31829b5b44 23877260

[19] Sharman JE, LaGerche A. Exercise blood pressure: clinical relevance and correct measurement. J Hum Hypertens 2015;29:351–8. 10.1038/jhh.2014.84 25273859

[20] Austin PC . Statistical power to detect violation of the proportional hazards assumption when using the Cox regression model. J Stat Comput Simul 2018;88:533–52. 10.1080/00949655.2017.1397151 29321694 PMC5758343

[21] Myers J, Prakash M, Froelicher V, Do D, Partington S, Atwood JE. Exercise capacity and mortality among men referred for exercise testing. N Engl J Med 2002;346:793–801. 10.1056/NEJMoa011858 11893790

[22] Kokkinos P, Faselis C, Samuel IBH, Pittaras A, Doumas M, Murphy R, et al Cardiorespiratory fitness and mortality risk across the Spectra of age, race, and sex. J Am Coll Cardiol 2022;80:598–609. 10.1016/j.jacc.2022.05.031 35926933

[23] Ross R, Blair SN, Arena R, Church TS, Despres JP, Franklin BA, et al Importance of assessing cardiorespiratory fitness in clinical practice: a case for fitness as a clinical vital sign: a scientific statement from the American Heart Association. Circulation 2016;134:e653–99. 10.1161/cir.0000000000000461 27881567

[24] Currie KD, Sasson Z, Goodman JM. Vascular-ventricular coupling during exercise is not affected by exaggerated blood pressures in endurance-trained athletes. J Appl Physiol (1985) 2019;127:753–9. 10.1152/japplphysiol.00108.2019 31318617

[25] Currie KD, Sless RT, Notarius CF, Thomas SG, Goodman JM. Absence of resting cardiovascular dysfunction in middle-aged endurance-trained athletes with exaggerated exercise blood pressure responses. J Hypertens 2017;35:1586–93. 10.1097/hjh.0000000000001365 28350576

[26] American College of Sports Medicine: Guidelines for Exercise Testing and prescription. 11th edn. Lippincott: Williams & Wilkins, 2022.

[27] Mancia G, Kreutz R, Brunström M, Burnier M, Grassi G, Januszewicz A, et al 2023 ESH guidelines for the management of arterial hypertension the task force for the management of arterial hypertension of the European society of hypertension: endorsed by the international society of hypertension (ISH) and the European renal association (ERA). J Hypertens 2023;41:1874–2071. 10.1097/hjh.0000000000003480 37345492

[28] Bouzas-Mosquera Mdel C, Bouzas-Mosquera A, Peteiro J, Broullón FJ, Alvarez-García N, Castro-Beiras A. Exaggerated exercise blood pressure response and risk of stroke in patients referred for stress testing. Eur J Intern Med 2014;25:533–7. 10.1016/j.ejim.2014.05.013 24930070

[29] Bouzas-Mosquera MC, Bouzas-Mosquera A, Peteiro J. Excessive blood pressure increase with exercise and risk of all-cause mortality and cardiac events. Eur J Clin Invest 2016;46:833–9. 10.1111/eci.12665 27505135

[30] Hedman K, Cauwenberghs N, Christle JW, Kuznetsova T, Haddad F, Myers J. Workload-indexed blood pressure response is superior to peak systolic blood pressure in predicting all-cause mortality. Eur J Prev Cardiol 2020;27:978–87. 10.1177/2047487319877268 31564136

[31] U.S. Department of Health and Human Services . The Surgeon General’s Call to Action to Control Hypertension. Washington, DC: U.S. Department of Health and Human Services, Office of the Surgeon General, 2020.

[32] Schutte AE, Webster R, Jennings G, Schlaich MP. Uncontrolled blood pressure in Australia: a call to action. Med J Aust 2022;216:61–3. 10.5694/mja2.51350 34865237

[33] Jones S, Schultz MG, Park C, Tillin T, Chaturvedi N, Hughes AD. Antihypertensive treatment effect on exercise blood pressure and exercise capacity in older adults. J Hypertens 2022;40:1682–91. 10.1097/hjh.0000000000003201 35881442

[34] Chant B, Bakali M, Hinton T, Burchell AE, Nightingale AK, Paton JFR, et al Antihypertensive treatment fails to control blood pressure during exercise. Hypertension 2018;72:102–9. 10.1161/hypertensionaha.118.11076 29895532

