# Supplementary material for: Exercise blood pressure relative to fitness and cardiovascular outcomes: the EXERTION study
Source: Eur Heart J. 2026 Jan 13;47(14):1661–71. doi: 10.1093/eurheartj/ehaf1082 (PMC13058452; doi:10.1093/eurheartj/ehaf1082)
Supplement: ehaf1082_Supplementary_Data [file ehaf1082_supplementary_data.docx]

**Table 1.** Basic demographic and exercise test data across groups with/without linked health data.

|  | Group 1  (Bruce/no linked data) | Group 2  (Bruce/linked data) | Group 3  (Final sample - Bruce/linked data + exclusions) |
| --- | --- | --- | --- |
| n | 5,323 | 24,754 | 12,733 |
| Age (years) | 55.0(14.6) | 55.2(13.8) | 52.7(13.0) |
| % Female Sex | 40.5 | 41.9 | 40.1 |
| Pre-exercise SBP | 130(18.9) n=1659 | 129(18.8) n=17565 | 127 (17.7) |
| Pre-exercise DBP | 78.3(11.3) n=1659 | 77.4(11.5) n=17565 | 75.9 (10.9) |
| Stage 1 SBP | 144(23.9) n=2327 | 148(23.5) n=15916 | 149(22.7) |
| Stage 2 SBP | 156(23.3) n=2670 | 163(26.3) n=16314 | 162(24.3) n=11339 |
| Stage 3 SBP | 163(24.2) n=1761 | 171(25.8) n=12838 | 172(23.9) n=9172 |
| Stage 4 SBP | 168(24.3) n=698 | 174 (25.6) n=7020 | 176(23.4) n=5216 |
| Peak SBP | 161(26.2) n=3989 | 179(26.9) n=18398 | 179 (25.1) |
| Peak METs | 9.69(3.30) n=3989 | 10.7(3.54) n=18389 | 11.1 (3.27) |

Data are mean and standard deviation or n (percentage). METs, metabolic equivalents. SBP, systolic blood pressure. DBP, diastolic blood pressure.

**Table 2.** Fatal and non-fatal event outcomes.

| **Event** | **n** |
| --- | --- |
| Cardiovascular events (total fatal + non-fatal) | 1,349 |
| Myocardial Infarction (121, 122) | 429 |
| Atrial Fibrillation (I48) | 635 |
| Heart Failure (I50) | 134 |
| Stroke (I60, I61, I63, I64) | 158 |
| Competing events (non-cardiovascular death) | 193 |

**Table 3.** Relative risk of fatal/non-fatal CVD events across quarters and 90^th^ percentile of SBP at each exercise test stage (all)

| **SBP/MET_Peak_** | **Model 1** | **Model 2** | **Model 3** | **Model 4** | **Model 5** | **Model 6** | **Model 7** |
| --- | --- | --- | --- | --- | --- | --- | --- |
|  | ***HR (95%CI)*** | ***HR (95%CI)*** | ***HR (95%CI)*** | ***HR (95%CI)*** | ***HR (95%CI)*** | ***HR (95%CI)*** | ***HR (95%CI)*** |
| **Stage 1 (n=12,596)** |  |  |  |  |  |  |  |
| 1^st^ Quarter | 1.00 (Ref) | 1.00 (Ref) | 1.00 (Ref) | 1.00 (Ref) | 1.00 (Ref) | 1.00 (Ref) | 1.00 (Ref) |
| 2^nd^ Quarter | 1.07 (0.91,1.25) | 0.88 (0.74,1.04) | 0.85 (0.72,1.01) | 0.85 (0.72,1.01) | 0.95 (0.70,1.30) | 0.81 (0.66,0.99) | 0.87 (0.58,1.30) |
| 3^rd^ Quarter | 1.11 (0.95,1.30) | 0.83 (0.70,0.97) | 0.78 (0.66,0.93) | 0.78 (0.66,0.93) | 0.89 (0.66,1.20) | 0.88 (0.72,1.07) | 0.74 (0.50,1.09) |
| 4^th^ Quarter | **1.48 (1.28,1.72)** | 0.86 (0.74,1.00) | 0.75 (0.64,0.87) | 0.78 (0.64,0.96) | 1.15 (0.87,1.52) | 0.90 (0.73,1.10) | 0.94 (0.65,1.35) |
| <90^th^ Percentile | 1.00 (Ref) | 1.00 (Ref) | 1.00 (Ref) | 1.00 (Ref) | 1.00 (Ref) | 1.00 (Ref) | 1.00 (Ref) |
| ≥90^th^ Percentile | **1.55 (1.31,1.83)** | 1.07 (0.90,1.26) | 0.91 (0.77,1.08) | 1.09 (0.91,1.31) | **1.30 (1.01,1.68)** | 1.10 (0.84,1.44) | 1.26 (0.91,1.74) |
| **Stage 2 (n=11,334)** |  |  |  |  |  |  |  |
| 1^st^ Quarter | 1.00 (Ref) | 1.00 (Ref) | 1.00 (Ref) | 1.00 (Ref) | 1.00 (Ref) | 1.00 (Ref) | 1.00 (Ref) |
| 2^nd^ Quarter | 1.02 (0.86,1.22) | 0.82 (0.69,0.98) | 0.78 (0.66,0.94) | 0.79 (0.66,0.95) | 1.05 (0.75,1.45) | 0.78 (0.63,0.97) | 0.79 (0.54,1.16) |
| 3^rd^ Quarter | 1.13 (0.95,1.34) | 0.81 (0.68,0.96) | 0.74 (0.62,0.88) | 0.76 (0.63,0.92) | 0.94 (0.67,1.31) | 0.87 (0.69,1.09) | 0.76 (0.53,1.08) |
| 4^th^ Quarter | **1.33 (1.11,1.59)** | 0.81 (0.67,0.97) | 0.69 (0.58,0.83) | 0.73 (0.58,0.92) | 1.09 (0.78,1.53) | 0.78 (0.60,1.03) | 0.86 (0.61,1.23) |
| <90^th^ Percentile | 1.00 (Ref) | 1.00 (Ref) | 1.00 (Ref) | 1.00 (Ref) | 1.00 (Ref) | 1.00 (Ref) | 1.00 (Ref) |
| ≥90^th^ Percentile | **1.26 (1.04,1.53)** | 0.96 (0.79,1.16) | 0.83 (0.69,1.01) | 0.96 (0.78,1.18) | 1.23 (0.91,1.67) | 0.87 (0.62,1.23) | 1.03 (0.75,1.41) |
| **Stage 3 (n=9,173)** |  |  |  |  |  |  |  |
| 1^st^ Quarter | 1.00 (Ref) | 1.00 (Ref) | 1.00 (Ref) | 1.00 (Ref) | 1.00 (Ref) | 1.00 (Ref) | 1.00 (Ref) |
| 2^nd^ Quarter | 0.94 (0.76,1.17) | 0.79 (0.64,0.98) | 0.78 (0.63,0.97) | 0.76 (0.61,0.95) | 1.25 (0.85,1.85) | 0.74 (0.57,0.96) | 0.64 (0.40,1.04) |
| 3^rd^ Quarter | 1.00 (0.80,1.25) | 0.74 (0.59,0.93) | 0.72 (0.57,0.91) | 0.70 (0.55,0.89) | 1.09 (0.72,1.67) | 0.78 (0.58,1.05) | 0.56 (0.35,0.89) |
| 4^th^ Quarter | 1.05 (0.85,1.30) | 0.71 (0.57,0.89) | 0.67 (0.54,0.83) | 0.65 (0.50,0.84) | 1.06 (0.70,1.59) | 0.65 (0.47,0.90) | 0.70 (0.46,1.07) |
| <90^th^ Percentile | 1.00 (Ref) | 1.00 (Ref) | 1.00 (Ref) | 1.00 (Ref) | 1.00 (Ref) | 1.00 (Ref) | 1.00 (Ref) |
| ≥90^th^ Percentile | **1.32 (1.04,1.68)** | 1.03 (0.81,1.31) | 0.97 (0.76,1.23) | 1.05 (0.81,1.36) | 1.34 (0.92,1.96) | 1.01 (0.67,1.53) | 1.16 (0.78,1.74) |
| **Stage 4 (n=5,210)** |  |  |  |  |  |  |  |
| 1^st^ Quarter | 1.00 (Ref) | 1.00 (Ref) | 1.00 (Ref) | 1.00 (Ref) | 1.00 (Ref) | 1.00 (Ref) | 1.00 (Ref) |
| 2^nd^ Quarter | 1.04 (0.74,1.47) | 0.87 (0.62,1.24) | 0.87 (0.62,1.24) | 0.86 (0.61,1.22) | 1.43 (0.75,2.73) | 0.68 (0.44,1.05) | 0.69 (0.33,1.41) |
| 3^rd^ Quarter | 0.87 (0.63,1.22) | 0.66 (0.47,0.93) | 0.66 (0.47,0.93) | 0.65 (0.46,0.93) | 1.40 (0.78,2.53) | 0.74 (0.50,1.11) | 0.67 (0.35,1.28) |
| 4^th^ Quarter | 1.05 (0.75,1.47) | 0.71 (0.50,1.01) | 0.70 (0.49,1.00) | 0.69 (0.47,1.01) | 1.37 (0.73,2.56) | 0.46 (0.26,0.81) | 0.74 (0.39,1.39) |
| <90^th^ Percentile | 1.00 (Ref) | 1.00 (Ref) | 1.00 (Ref) | 1.00 (Ref) | 1.00 (Ref) | 1.00 (Ref) | 1.00 (Ref) |
| ≥90^th^ Percentile | 1.29 (0.81,2.03) | 1.01 (0.64,1.60) | 0.98 (0.62,1.56) | 1.08 (0.67,1.74) | 1.43 (0.69,2.94) | 0.81 (0.33,1.97) | 1.22 (0.59,2.54) |
| **Peak (12,690)** |  |  |  |  |  |  |  |
| 1^st^ Quarter | 1.00 (Ref) | 1.00 (Ref) | 1.00 (Ref) | 1.00 (Ref) | 1.00 (Ref) | 1.00 (Ref) | 1.00 (Ref) |
| 2^nd^ Quarter | 0.73 (0.64,0.85) | 0.67 (0.58,0.77) | 0.75 (0.65,0.87) | 0.63 (0.54,0.73) | 0.79 (0.61,1.02) | 0.65 (0.54,0.77) | 0.61 (0.43,0.86) |
| 3^rd^ Quarter | 0.87 (0.74,1.02) | 0.70 (0.60,0.82) | 0.81 (0.69,0.96) | 0.63 (0.53,0.75) | 0.98 (0.75,1.29) | 0.75 (0.60,0.94) | 0.64 (0.45,0.91) |
| 4^th^ Quarter | 0.91 (0.79,1.05) | 0.67 (0.58,0.77) | 0.75 (0.64,0.86) | 0.57 (0.48,0.67) | 0.90 (0.70,1.17) | 0.64 (0.52,0.80) | 0.57 (0.41,0.81) |
| <90^th^ Percentile | 1.00 (Ref) | 1.00 (Ref) | 1.00 (Ref) | 1.00 (Ref) | 1.00 (Ref) | 1.00 (Ref) | 1.00 (Ref) |
| ≥90^th^ Percentile | **1.18 (1.00,1.41)** | 0.95 (0.80,1.13) | 0.95 (0.80,1.13) | 0.93 (0.78,1.12) | 1.09 (0.82,1.45) | 0.76 (0.56,1.04) | 0.98 (0.71,1.34) |

Model 1 is univariable. Model 2 is adjusted for age and sex. Model 3 is adjusted for age, sex and fitness. Model 4 is adjusted for age, sex and pre-exercise systolic BP. Model 5 is adjusted for age and sex and limited to those with no CVD history (n=2,145 – 5,618 across stages). Model 6 is adjusted for age and sex and limited to those with a pre-exercise systolic blood pressure <140/90 mmHg (n=3,408 – 7,776 across stages). Model 7 is adjusted for age, sex and potential blood pressure lowering medication (yes/no), limited to those with medications reported (n=1,016 – 2,491 across stages). Bold data indicate statistical significance. Quartile 1 is the reference group (Ref).

**Table 4.** Relative risk of fatal/non-fatal CVD events in males across quarters and 90th percentile of SBP/MET_Peak_ at each exercise test stage.

| **SBP/MET_Peak_** | **Model 1** | **Model 2** | **Model 3** | **Model 4** | **Model 5** | **Model 6** |
| --- | --- | --- | --- | --- | --- | --- |
|  | ***HR (95%CI)*** | ***HR (95%CI)*** | ***HR (95%CI)*** | ***HR (95%CI)*** | ***HR (95%CI)*** | ***HR (95%CI)*** |
| **Stage 1 (n=7,604)** |  |  |  |  |  |  |
| 1^st^ Quarter | 1.00 (Ref) | 1.00 (Ref) | 1.00 (Ref) | 1.00 (Ref) | 1.00 (Ref) | 1.00 (Ref) |
| 2^nd^ Quarter | **1.33 (1.07,1.66)** | 1.05 (0.84,1.31) | 1.10 (0.88,1.38) | 0.87 (0.58,1.28) | 1.03 (0.79,1.36) | 0.97 (0.60,1.55) |
| 3^rd^ Quarter | **2.01 (1.63,2.47)** | **1.32 (1.06,1.65)** | **1.44 (1.15,1.81)** | 1.20 (0.82,1.73) | **1.45 (1.11,1.89)** | 1.04 (0.66,1.65) |
| 4^th^ Quarter | **4.13 (3.41,4.99)** | **2.08 (1.67,2.59)** | **2.36 (1.88,2.97)** | **1.62 (1.11,2.36)** | **2.17 (1.66,2.84)** | 1.34 (0.83,2.15) |
| <90^th^ Percentile | 1.00 (Ref) | 1.00 (Ref) | 1.00 (Ref) | 1.00 (Ref) | 1.00 (Ref) | 1.00 (Ref) |
| ≥90th Percentile | **3.24 (2.76,3.80)** | **1.79 (1.50,2.13)** | **1.87 (1.56,2.25)** | **1.85 (1.36,2.52)** | **2.14 (1.68,2.72)** | **1.61 (1.03,2.51)** |
| **Stage 2 (n=6,896)** |  |  |  |  |  |  |
| 1^st^ Quarter | 1.00 (Ref) | 1.00 (Ref) | 1.00 (Ref) | 1.00 (Ref) | 1.00 (Ref) | 1.00 (Ref) |
| 2^nd^ Quarter | **1.42 (1.12,1.80)** | 1.11 (0.87,1.41) | 1.15 (0.90,1.46) | 0.75 (0.49,1.15) | 1.24 (0.93,1.66) | 1.10 (0.66,1.82) |
| 3^rd^ Quarter | **1.93 (1.54,2.43)** | **1.29 (1.01,1.64)** | **1.37 (1.07,1.75)** | 1.11 (0.74,1.66) | **1.38 (1.02,1.88)** | 1.16 (0.72,1.90) |
| 4^th^ Quarter | **3.37 (2.72,4.17)** | **1.78 (1.40,2.26)** | **1.95 (1.52,2.51)** | 1.37 (0.91,2.08) | **1.81 (1.33,2.46)** | 1.35 (0.83,2.20) |
| <90^th^ Percentile | 1.00 (Ref) | 1.00 (Ref) | 1.00 (Ref) | 1.00 (Ref) | 1.00 (Ref) | 1.00 (Ref) |
| ≥90^th^ Percentile | **2.47 (2.04,3.00)** | **1.46 (1.19,1.79)** | **1.51 (1.22,1.86)** | **1.40 (0.96,2.03)** | **1.45 (1.05,1.99)** | 1.32 (0.93,1.89) |
| **Stage 3 (n=5,738)** |  |  |  |  |  |  |
| 1^st^ Quarter | 1.00 (Ref) | 1.00 (Ref) | 1.00 (Ref) | 1.00 (Ref) | 1.00 (Ref) | 1.00 (Ref) |
| 2^nd^ Quarter | **1.41 (1.07,1.86)** | 1.14 (0.86,1.51) | 1.17 (0.88,1.55) | 1.14 (0.71,1.84) | 1.28 (0.91,1.79) | 1.15 (0.64,2.06) |
| 3^rd^ Quarter | **1.77 (1.35,2.32)** | 1.25 (0.94,1.66) | 1.30 (0.98,1.73) | 1.24 (0.74,2.07) | 1.31 (0.91,1.88) | 1.04 (0.57,1.87) |
| 4^th^ Quarter | **2.47 (1.91,3.20)** | **1.52 (1.14,2.03)** | **1.63 (1.21,2.20)** | 1.56 (0.95,2.58) | 1.46 (0.99,2.15) | 1.00 (0.55,1.79) |
| <90^th^ Percentile | 1.00 (Ref) | 1.00 (Ref) | 1.00 (Ref) | 1.00 (Ref) | 1.00 (Ref) | 1.00 (Ref) |
| ≥90^th^ Percentile | **2.21 (1.72,2.83)** | **1.54 (1.19,2.00)** | **1.61 (1.23,2.11)** | **1.75 (1.07,2.87)** | 1.50 (0.97,2.31) | 1.33 (0.85,2.10) |
| **Stage 4 (n=3,536)** |  |  |  |  |  |  |
| 1^st^ Quarter | 1.00 (Ref) | 1.00 (Ref) | 1.00 (Ref) | 1.00 (Ref) | 1.00 (Ref) | 1.00 (Ref) |
| 2^nd^ Quarter | 1.14 (0.77,1.70) | 0.96 (0.65,1.42) | 0.98 (0.66,1.47) | 1.02 (0.50,2.09) | 0.98 (0.62,1.56) | 0.89 (0.41,1.96) |
| 3^rd^ Quarter | 1.19 (0.80,1.77) | 0.83 (0.55,1.23) | 0.87 (0.57,1.31) | 1.16 (0.60,2.26) | 0.86 (0.52,1.41) | 0.57 (0.25,1.30) |
| 4^th^ Quarter | **1.73 (1.18,2.54)** | 0.99 (0.66,1.47) | 1.06 (0.70,1.61) | 1.76 (0.89,3.48) | 0.84 (0.48,1.48) | 0.78 (0.35,1.77) |
| <90^th^ Percentile | 1.00 (Ref) | 1.00 (Ref) | 1.00 (Ref) | 1.00 (Ref) | 1.00 (Ref) | 1.00 (Ref) |
| ≥90^th^ Percentile | **1.90 (1.29,2.81)** | 1.26 (0.84,1.89) | 1.34 (0.88,2.04) | 1.91 (0.91,4.03) | 1.19 (0.62,2.27) | 0.84 (0.39,1.80) |
| **Peak (n=7,615)** |  |  |  |  |  |  |
| 1^st^ Quarter | 1.00 (Ref) | 1.00 (Ref) | 1.00 (Ref) | 1.00 (Ref) | 1.00 (Ref) | 1.00 (Ref) |
| 2^nd^ Quarter | **1.37 (1.10,1.72)** | 1.04 (0.83,1.31) | 1.08 (0.86,1.36) | 0.98 (0.66,1.47) | 0.97 (0.74,1.28) | 0.74 (0.45,1.21) |
| 3^rd^ Quarter | **2.27 (1.84,2.79)** | **1.48 (1.19,1.84)** | **1.58 (1.27,1.97)** | **1.56 (1.07,2.27)** | **1.46 (1.12,1.91)** | 0.95 (0.60,1.52) |
| 4^th^ Quarter | **4.08 (3.36,4.96)** | **2.01 (1.61,2.51)** | **2.24 (1.78,2.81)** | **1.80 (1.22,2.66)** | **2.02 (1.54,2.65)** | 1.12 (0.68,1.82) |
| <90^th^ Percentile | 1.00 (Ref) | 1.00 (Ref) | 1.00 (Ref) | 1.00 (Ref) | 1.00 (Ref) | 1.00 (Ref) |
| ≥90^th^ Percentile | **2.92 (2.49,3.44)** | **1.61 (1.34,1.92)** | **1.67 (1.39,2.01)** | **1.51 (1.10,2.09)** | **1.93 (1.52,2.46)** | 1.31 (0.85,2.02) |

Model 1 is univariable. Model 2 is adjusted for age. Model 3 is adjusted for age and pre-exercise systolic BP. Model 4 is adjusted for age and limited to those with no CVD history (n=1,393 – 3,313 across stages). Model 5 is adjusted for age and limited to those with a pre-exercise systolic blood pressure <130/90 mmHg (n=2,167 – 4,470 across stages). Model 6 is adjusted for age and potential blood pressure lowering medication (yes/no), limited to those with current medications reported (n=658 – 1,441 across stages). Bold data indicate statistical significance.

**Table 5.** Relative risk of fatal/non-fatal CVD event in females across quarters and 90th percentile of SBP/MET_Peak_ at each exercise test stage.

| **SBP/MET_Peak_** | **Model 1** | **Model 2** | **Model 3** | **Model 4** | **Model 5** | **Model 6** |
| --- | --- | --- | --- | --- | --- | --- |
|  | ***HR (95%CI)*** | ***HR (95%CI)*** | ***HR (95%CI)*** | ***HR (95%CI)*** | ***HR (95%CI)*** | ***HR (95%CI)*** |
| **Stage 1 (n=5,082)** |  |  |  |  |  |  |
| 1^st^ Quarter | 1.00 (Ref) | 1.00 (Ref) | 1.00 (Ref) | 1.00 (Ref) | 1.00 (Ref) | 1.00 (Ref) |
| 2^nd^ Quarter | **1.66 (1.04,2.63)** | 1.33 (0.83,2.13) | 1.38 (0.86,2.21) | 1.04 (0.52,2.07) | 1.24 (0.73,2.10) | 1.23 (0.46,3.25) |
| 3^rd^ Quarter | **2.00 (1.29,3.10)** | 1.37 (0.87,2.16) | 1.46 (0.92,2.31) | 1.17 (0.61,2.23) | 1.57 (0.95,2.61) | 1.13 (0.43,2.98) |
| 4^th^ Quarter | **5.67 (3.81,8.44)** | **3.00 (1.93,4.65)** | **3.33 (2.10,5.27)** | **2.13 (1.14,3.97)** | **3.43 (2.09,5.64)** | 2.58 (0.97,6.81) |
| <90^th^ Percentile | 1.00 (Ref) | 1.00 (Ref) | 1.00 (Ref) | 1.00 (Ref) | 1.00 (Ref) | 1.00 (Ref) |
| ≥90^th^ Percentile | **3.36 (2.72,4.16)** | **1.97 (1.54,2.53)** | **2.03 (1.55,2.66)** | **2.05 (1.45,2.90)** | **2.35 (1.64,3.35)** | 1.75 (0.97,3.17) |
| **Stage 2 (n=4,453)** |  |  |  |  |  |  |
| 1^st^ Quarter | 1.00 (Ref) | 1.00 (Ref) | 1.00 (Ref) | 1.00 (Ref) | 1.00 (Ref) | 1.00 (Ref) |
| 2^nd^ Quarter | 1.46 (0.89,2.39) | 1.18 (0.72,1.95) | 1.21 (0.74,2.00) | 1.29 (0.62,2.69) | 1.11 (0.63,1.95) | 0.69 (0.27,1.78) |
| 3^rd^ Quarter | **1.89 (1.20,2.98)** | 1.35 (0.84,2.17) | 1.42 (0.88,2.29) | 1.35 (0.66,2.76) | 1.38 (0.81,2.36) | 0.67 (0.28,1.64) |
| 4^th^ Quarter | **3.64 (2.37,5.59)** | **2.19 (1.36,3.51)** | **2.37 (1.43,3.91)** | 1.82 (0.89,3.73) | **2.72 (1.59,4.66)** | 1.35 (0.54,3.37) |
| <90^th^ Percentile | 1.00 (Ref) | 1.00 (Ref) | 1.00 (Ref) | 1.00 (Ref) | 1.00 (Ref) | 1.00 (Ref) |
| ≥90^th^ Percentile | **2.63 (2.00,3.46)** | **1.79 (1.32,2.44)** | **1.85 (1.33,2.58)** | **1.51 (0.91,2.51)** | **2.29 (1.50,3.49)** | 1.58 (0.92,2.72) |
| **Stage 3 (n=3,437)** |  |  |  |  |  |  |
| 1^st^ Quarter | 1.00 (Ref) | 1.00 (Ref) | 1.00 (Ref) | 1.00 (Ref) | 1.00 (Ref) | 1.00 (Ref) |
| 2^nd^ Quarter | 1.55 (0.84,2.85) | 1.18 (0.63,2.21) | 1.19 (0.64,2.23) | 1.51 (0.60,3.81) | 1.12 (0.54,2.31) | 0.54 (0.19,1.56) |
| 3^rd^ Quarter | **2.33 (1.33,4.11)** | 1.60 (0.88,2.90) | 1.64 (0.90,3.00) | 1.84 (0.76,4.42) | 1.71 (0.87,3.36) | 0.43 (0.15,1.26) |
| 4^th^ Quarter | **2.64 (1.51,4.64)** | 1.49 (0.80,2.77) | 1.55 (0.80,3.00) | 1.41 (0.54,3.67) | 2.02 (0.98,4.14) | 0.43 (0.14,1.34) |
| <90^th^ Percentile | 1.00 (Ref) | 1.00 (Ref) | 1.00 (Ref) | 1.00 (Ref) | 1.00 (Ref) | 1.00 (Ref) |
| ≥90^th^ Percentile | **2.08 (1.37,3.17)** | 1.40 (0.89,2.20) | 1.41 (0.86,2.32) | 0.93 (0.40,2.16) | **2.09 (1.08,4.07)** | 1.07 (0.43,2.67) |
| **Stage 4 (n=1,680)** |  |  |  |  |  |  |
| 1^st^ Quarter | 1.00 (Ref) | 1.00 (Ref) | 1.00 (Ref) | 1.00 (Ref) | 1.00 (Ref) | 1.00 (Ref) |
| 2^nd^ Quarter | 1.01 (0.42,2.43) | 0.75 (0.30,1.88) | 0.74 (0.29,1.86) | 1.06 (0.30,3.67) | 0.86 (0.30,2.45) | 0.07 (0.01,0.59) |
| 3^rd^ Quarter | 1.47 (0.66,3.30) | 0.99 (0.43,2.27 | 0.95 (0.40,2.23) | 0.82 (0.23,2.92) | 1.12 (0.41,3.05) | 0.43 (0.13,1.41) |
| 4^th^ Quarter | 1.36 (0.60,3.05) | 0.66 (0.29,1.50) | 0.62 (0.26,1.52) | 0.79 (0.24,2.61) | 0.83 (0.31,2.19) | 0.14 (0.03,0.74) |
| <90^th^ Percentile | 1.00 (Ref) | 1.00 (Ref) | 1.00 (Ref) | 1.00 (Ref) | 1.00 (Ref) | 1.00 (Ref) |
| ≥90^th^ Percentile | **2.12 (1.03,4.37)** | 1.26 (0.62,2.55) | 1.27 (0.59,2.72) | 1.33 (0.47,3.74) | 1.46 (0.57,3.76) | 0.64 (0.14,2.99) |
| **Peak (n=5,102)** |  |  |  |  |  |  |
| 1^st^ Quarter | 1.00 (Ref) | 1.00 (Ref) | 1.00 (Ref) | 1.00 (Ref) | 1.00 (Ref) | 1.00 (Ref) |
| 2^nd^ Quarter | **1.83 (1.17,2.87)** | 1.45 (0.92,2.28) | 1.48 (0.95,2.33) | 1.65 (0.84,3.23) | 1.32 (0.80,2.20) | 1.11 (0.46,2.67) |
| 3^rd^ Quarter | **2.34 (1.53,3.60)** | **1.58 (1.02,2.46)** | **1.65 (1.06,2.57)** | 1.68 (0.86,3.28) | **1.69 (1.03,2.75)** | 1.05 (0.45,2.48) |
| 4^th^ Quarter | **5.78 (3.90,8.55)** | **2.99 (1.95,4.59)** | **3.23 (2.08,5.02)** | **2.67 (1.40,5.08)** | **3.20 (1.97,5.17)** | 2.16 (0.93,5.03) |
| <90^th^ Percentile | 1.00 (Ref) | 1.00 (Ref) | 1.00 (Ref) | 1.00 (Ref) | 1.00 (Ref) | 1.00 (Ref) |
| ≥90^th^ Percentile | **2.95 (2.37,3.68)** | **2.95 (2.37,3.68)** | **1.70 (1.30,2.24)** | **1.49 (1.05,2.13)** | **2.15 (1.50,3.09)** | 1.09 (0.59,1.98) |

Model 1 is univariable. Model 2 is adjusted for age. Model 3 is adjusted for age and pre-exercise systolic BP. Model 4 is adjusted for age and limited to those with no CVD history (n=754 – 2,514 across stages). Model 5 is adjusted for age and limited to those with a pre-exercise systolic blood pressure <130/90 mmHg (n=1,247- 3,334 across stages). Model 6 is adjusted for age and potential blood pressure lowering medication (yes/no), limited to those with current medications reported (n=359 – 1,051 across stages). Bold data indicate statistical significance.

**Table 6.** Relative risk of fatal/non-fatal CVD event at various cut-points of SBP/MET_Peak_ across each exercise test stage amongst study subgroups.

|  | ≥15 mmHg/MET_Peak_  *HR (95%CI)* | | | ≥18 mmHg/MET_Peak_  *HR (95%CI)* | | | ≥20 mmHg/MET_Peak_  *HR (95%CI)* | | |
| --- | --- | --- | --- | --- | --- | --- | --- | --- | --- |
| ***All**** | Model 1 | Model 2 | Model 3 | Model 1 | Model 2 | Model 3 | Model 1 | Model 2 | Model 3 |
| Stage 1 | **1.73 (1.40,2.13)** | **2.10 (1.79,2.45)** | **1.34 (1.04,1.73)** | **1.65 (1.34,2.03)** | **1.96 (1.66,2.31)** | **1.40 (1.08,1.83)** | **1.76 (1.42,2.19)** | **2.05 (1.72,2.44)** | **1.55 (1.16,2.06)** |
| Stage 2 | **1.43 (1.12,1.81)** | **1.57 (1.32,1.87)** | 1.26 (0.96,1.65) | **1.42 (1.11,1.81)** | **1.69 (1.40,2.04)** | **1.38 (1.07,1.79)** | **1.37 (1.05,1.78)** | **1.49 (1.19,1.87)** | **1.34 (1.02,1.76)** |
| Stage 3 | 1.22 (0.92,1.62) | 1.17 (0.94,1.46) | 0.87 (0.63,1.21) | 1.31 (0.92,1.85) | 1.51 (1.13,2.00) | 0.92 (0.64,1.33) | **1.57 (1.02,2.42)** | **1.56 (1.05,2.30)** | 1.28 (0.84,1.94) |
| Stage 4 | 1.37 (0.84,2.24) | 0.88 (0.58,1.34) | 0.89 (0.53,1.51) | **1.99 (1.02,3.91)** | 1.54 (0.85,2.78) | 0.77 (0.35,1.69) | 2.25 (0.91,5.53) | 1.30 (0.50,3.40) | 0.45 (0.11,1.84) |
| Peak | **1.33 (1.06,1.68)** | **1.52 (1.29,1.80)** | 1.05 (0.78,1.41) | **1.65 (1.34,2.04)** | **2.01 (1.71,2.36)** | **1.33 (1.02,1.73)** | **1.54 (1.25,1.90)** | **1.85 (1.57,2.17)** | **1.37 (1.07,1.77)** |
| ***Female*** |  |  |  |  |  |  |  |  |  |
| Stage 1 | **1.90 (1.32,2.73)** | **2.50 (1.86,3.37)** | **1.88 (1.13,3.15)** | 1.86 (1.32,2.60) | 2.35 (1.75,3.17) | 2.21 (1.34,3.65) | **1.80 (1.29,2.53)** | **2.26 (1.66,3.09)** | **1.87 (1.12,3.12)** |
| Stage 2 | **1.41 (0.90,2.20)** | **1.82 (1.30,2.54)** | 1.20 (0.70,2.03) | 1.42 (0.93,2.18) | 2.26 (1.60,3.17) | 1.89 (1.14,3.14) | 1.49 (0.93,2.36) | **2.05 (1.39,3.02)** | **1.78 (1.07,2.97)** |
| Stage 3 | 1.11 (0.67,1.86) | 1.29 (0.84,1.96) | 0.59 (0.30,1.17) | 0.86 (0.44,1.68) | 1.70 (0.98,2.93) | 0.82 (0.36,1.87) | 1.05 (0.45,2.46) | **2.25 (1.12,4.52)** | 1.04 (0.39,2.79) |
| Stage 4 | 0.84 (0.34,2.05) | 0.84 (0.40,1.77) | 0.46 (0.14,1.56) | 1.90 (0.67,5.42) | 2.46 (0.95,6.37) | 0.42 (0.06,2.98) | 2.56 (0.68,9.67) | 2.95 (0.61,14.35) | N/A |
| Peak | 1.32 (0.87,2.00) | **1.62 (1.17,2.23)** | 1.07 (0.60,1.89) | 1.58 (1.10,2.26) | 2.46 (0.95,6.37) | 1.70 (1.00,2.91) | **1.62 (1.15,2.29)** | **2.09 (1.55,2.81)** | 1.93 (1.20,3.11) |
| ***Male*** |  |  |  |  |  |  |  |  |  |
| Stage 1 | **1.64 (1.27,2.13)** | **1.95 (1.61,2.35)** | 1.17 (0.87,1.58) | 1.53 (1.17,2.00) | 1.79 (1.46,2.20) | 1.14 (0.83,1.57) | **1.73 (1.31,2.29)** | **1.95 (1.58,2.41)** | 1.40 (0.99,1.98) |
| Stage 2 | **1.44 (1.09,1.90)** | **1.48 (1.20,1.83)** | 1.28 (0.94,1.75) | 1.43 (1.07,1.92) | 1.47 (1.17,1.85) | 1.21 (0.90,1.63) | 1.33 (0.96,1.83) | 1.27 (0.96,1.68) | 1.17 (0.85,1.62) |
| Stage 3 | 1.26 (0.90,1.77) | 1.14 (0.88,1.47) | 0.97 (0.67,1.40) | 1.54 (1.03,2.30) | 1.47 (1.17,1.85) | 0.95 (0.63,1.44) | **1.86 (1.13,3.05)** | 1.35 (0.84,2.17) | 1.36 (0.86,2.15) |
| Stage 4 | 1.64 (0.93,2.89) | 0.90 (0.55,1.46) | 1.05 (0.59,1.88) | 2.02 (0.86,4.78) | 1.32 (0.64,2.74) | 0.89 (0.38,2.09) | 1.96 (0.57,6.75) | 0.94 (0.28,3.16) | 0.69 (0.17,2.75) |
| Peak | **1.34 (1.01,1.78)** | **1.49 (1.22,1.82)** | 1.04 (0.73,1.48) | 1.69 (1.30,2.19) | 1.96 (1.62,2.37) | 1.21 (0.89,1.63) | **1.49 (1.15,1.94)** | **1.75 (1.44,2.12)** | 1.18 (0.88,1.60) |

***Table 6 continued.***

| ≥24 mmHg/MET_Peak_  *HR (95%CI)* | | |
| --- | --- | --- |
| Model 1 | Model 2 | Model 3 |
| **1.91 (1.52,2.41)** | **2.15 (1.77,2.63)** | **1.72 (1.21,2.44)** |
| **1.51 (1.10,2.06)** | **1.68 (1.27,2.24)** | **1.53 (1.12,2.09)** |
| **1.98 (1.02,3.85)** | **3.31 (1.91,5.75)** | 1.40 (0.70,2.80) |
| **4.09 (1.10,15.18)** | 2.09 (0.41,10.58) | 0.52 (0.07,3.92) |
| **1.68 (1.35,2.09)** | **1.98 (1.65,2.37)** | 1.53 (1.14,2.05) |
|  |  |  |
| **2.01 (1.42,2.85)** | **2.22 (1.55,3.18)** | 1.79 (0.98,3.25) |
| 1.30 (0.75,2.24) | 1.81 (1.10,2.98) | 1.38 (0.77,2.48) |
| 1.50 (0.45,5.01) | **6.59 (2.82,15.39)** | 0.40 (0.05,3.31) |
| 4.40 (0.46,42.12) | **21.98 (1.89,255.48)** | N/A |
| **1.73 (1.24,2.42)** | **2.20 (1.59,3.04)** | **1.93 (1.15,3.23)** |
|  |  |  |
| **1.84 (1.35,2.51)** | **2.12 (1.66,2.69)** | **1.67 (1.08,2.60)** |
| **1.66 (1.13,2.44)** | **1.62 (1.14,2.29)** | **1.60 (1.11,2.32)** |
| **2.28 (1.04,5.03)** | **2.23 (1.07,4.65)** | 2.03 (0.98,4.21) |
| .65 (0.84,15.84) | 1.07 (0.13,9.11) | 0.88 (0.12,6.67) |
| **1.64 (1.23,2.20)** | **1.87 (1.50,2.33)** | 1.35 (0.94,1.94) |

Data are hazard ratios and 95% confidence intervals adjusted for age and pre-exercise SBP. *Models also adjusted for sex. Model 1 is limited to those with a pre-exercise systolic blood pressure <130/90 mmHg. Model 2 is limited to those with no CVD history. Model 3 is limited to those with medications reported and additionally adjusted for use of BP lowering medication (yes/no). N/A, data not available due to insufficient numbers for the model to converge. Reference group is < each cut-point value.

**Table 7.** Number of patients at risk (Data corresponding to Figure 2A).

|  | Q1 | | | Q2 | | | Q3 | | | Q4 | | |
| --- | --- | --- | --- | --- | --- | --- | --- | --- | --- | --- | --- | --- |
| Time (months) | Number at risk | CVD event | Competing event | Number at risk | CVD event | Competing event | Number at risk | CVD event | Competing event | Number at risk | CVD event | Competing event |
| 0 | 3200 | 0 | 0 | 3143 | 0 | 0 | 3171 | 0 | 0 | 3172 | 0 | 0 |
| 25 | 2767 | 72 | 4 | 2659 | 113 | 10 | 2672 | 130 | 19 | 2478 | 309 | 28 |
| 50 | 1729 | 41 | 4 | 1685 | 56 | 14 | 1643 | 87 | 14 | 1454 | 159 | 29 |
| 75 | 853 | 33 | 4 | 780 | 35 | 5 | 796 | 42 | 6 | 707 | 89 | 15 |
| 100 | 391 | 10 | 2 | 431 | 11 | 5 | 451 | 28 | 3 | 446 | 36 | 10 |
| 125 | 120 | 11 | 1 | 181 | 9 | 4 | 189 | 12 | 4 | 153 | 35 | 8 |
| 150 | 11 | 3 | 0 | 21 | 3 | 0 | 22 | 11 | 1 | 15 | 8 | 2 |

**Table 8.** Number of patients at risk (Data corresponding to Figure 2B).

|  | Q1 | | | Q2 | | | Q3 | | | Q4 | | |
| --- | --- | --- | --- | --- | --- | --- | --- | --- | --- | --- | --- | --- |
| Time (months) | Number at risk | CVD event | Competing event | Number at risk | CVD event | Competing event | Number at risk | CVD event | Competing event | Number at risk | CVD event | Competing event |
| 0 | 3289 | 0 | 0 | 2985 | 0 | 0 | 3221 | 0 | 0 | 3101 | 0 | 0 |
| 25 | 2800 | 159 | 10 | 2476 | 142 | 17 | 2656 | 142 | 13 | 2567 | 176 | 21 |
| 50 | 1716 | 71 | 14 | 1531 | 63 | 12 | 1625 | 91 | 14 | 1594 | 116 | 20 |
| 75 | 841 | 35 | 5 | 718 | 48 | 5 | 786 | 51 | 10 | 764 | 65 | 10 |
| 100 | 443 | 16 | 3 | 390 | 18 | 4 | 406 | 18 | 6 | 469 | 33 | 7 |
| 125 | 161 | 16 | 2 | 121 | 10 | 3 | 170 | 12 | 4 | 188 | 29 | 8 |
| 150 | 16 | 5 | 0 | 13 | 4 | 0 | 21 | 9 | 1 | 19 | 7 | 2 |

**Table 9.** Number of patients at risk (Data corresponding to Figure 2C).

|  | <90^th^ percentile | | | >90th percentile | | |
| --- | --- | --- | --- | --- | --- | --- |
| Time (months) | Number at risk | CVD event | Competing event | Number at risk | CVD event | Competing event |
| 0 | 11417 | 0 | 0 | 1269 | 0 | 0 |
| 25 | 9629 | 461 | 46 | 947 | 163 | 15 |
| 50 | 5966 | 274 | 50 | 545 | 69 | 11 |
| 75 | 2858 | 156 | 25 | 278 | 43 | 5 |
| 100 | 1525 | 64 | 14 | 194 | 21 | 6 |
| 125 | 583 | 48 | 12 | 60 | 19 | 5 |
| 150 | 61 | 21 | 2 | 8 | 4 | 1 |

**Table 10.** Number of patients at risk (Data corresponding to Figure 2D).

|  | <90th percentile | | | >90th percentile | | |
| --- | --- | --- | --- | --- | --- | --- |
| Time (months) | Number at risk | CVD event | Competing event | Number at risk | CVD event | Competing event |
| 0 | 11596 | 0 | 0 | 1000 | 0 | 0 |
| 25 | 9687 | 556 | 54 | 812 | 63 | 7 |
| 50 | 5978 | 290 | 56 | 488 | 51 | 4 |
| 75 | 2864 | 175 | 29 | 245 | 24 | 1 |
| 100 | 1543 | 77 | 18 | 165 | 8 | 2 |
| 125 | 575 | 55 | 15 | 65 | 12 | 2 |
| 150 | 62 | 25 | 3 | 7 | 0 | 0 |

**Table 11.** Number of patients at risk (Data corresponding to Figure 2A).

|  | <18 mmHg/MET_Peak_ | | | >18 mmHg/MET_Peak_ | | |
| --- | --- | --- | --- | --- | --- | --- |
| Time (months) | Number at risk | CVD event | Competing event | Number at risk | CVD event | Competing event |
| 0 | 9732 | 0 | 0 | 2954 | 0 | 0 |
| 25 | 8269 | 337 | 36 | 2307 | 287 | 25 |
| 50 | 5160 | 195 | 32 | 1351 | 148 | 29 |
| 75 | 2474 | 112 | 15 | 662 | 87 | 15 |
| 100 | 1293 | 50 | 11 | 426 | 35 | 9 |
| 125 | 494 | 34 | 9 | 149 | 33 | 8 |
| 150 | 54 | 17 | 1 | 15 | 8 | 2 |


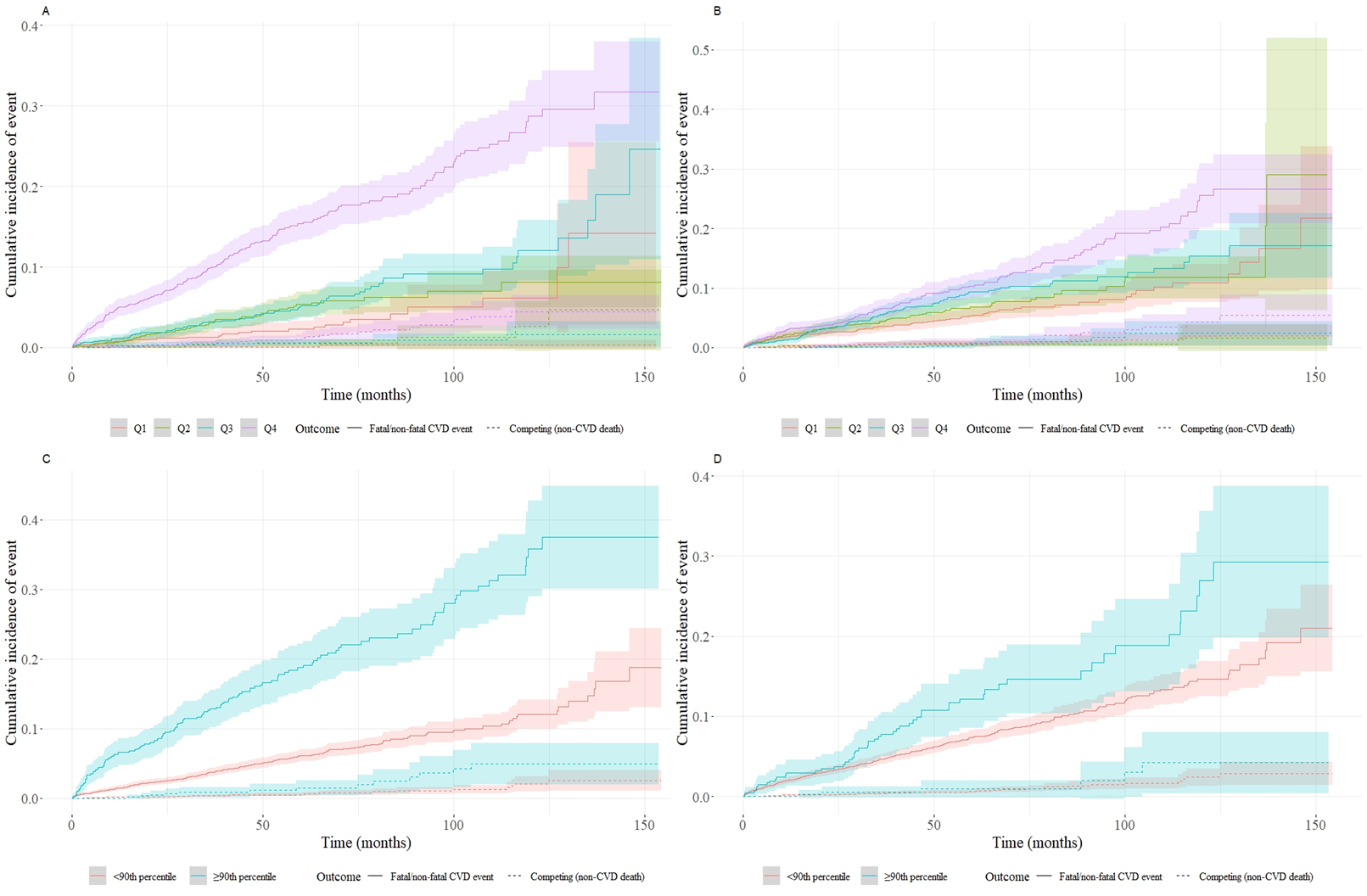


**Supplementary Figure 1 (Females)**

**
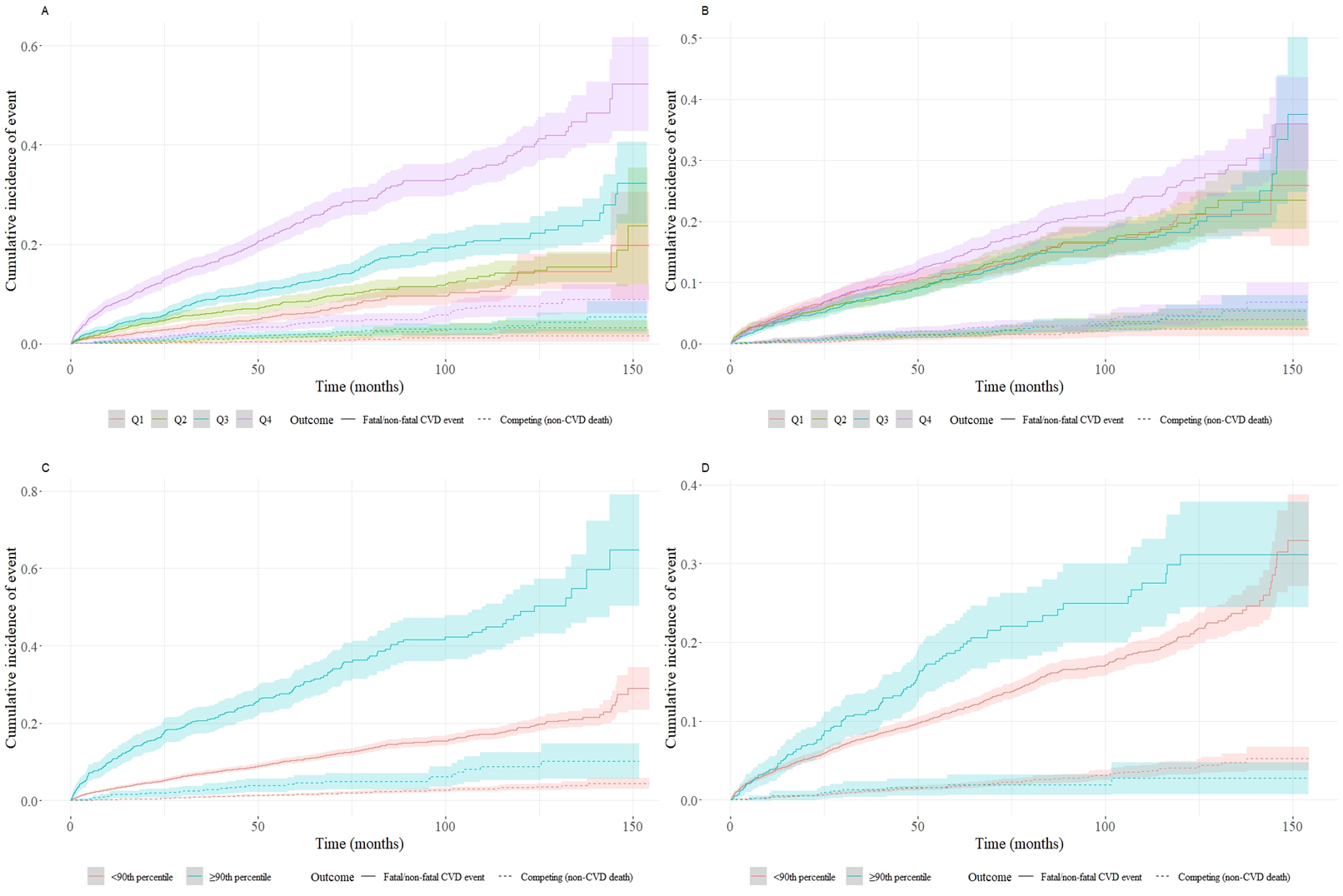
**

**Supplementary Figure 2 (Males)**

**
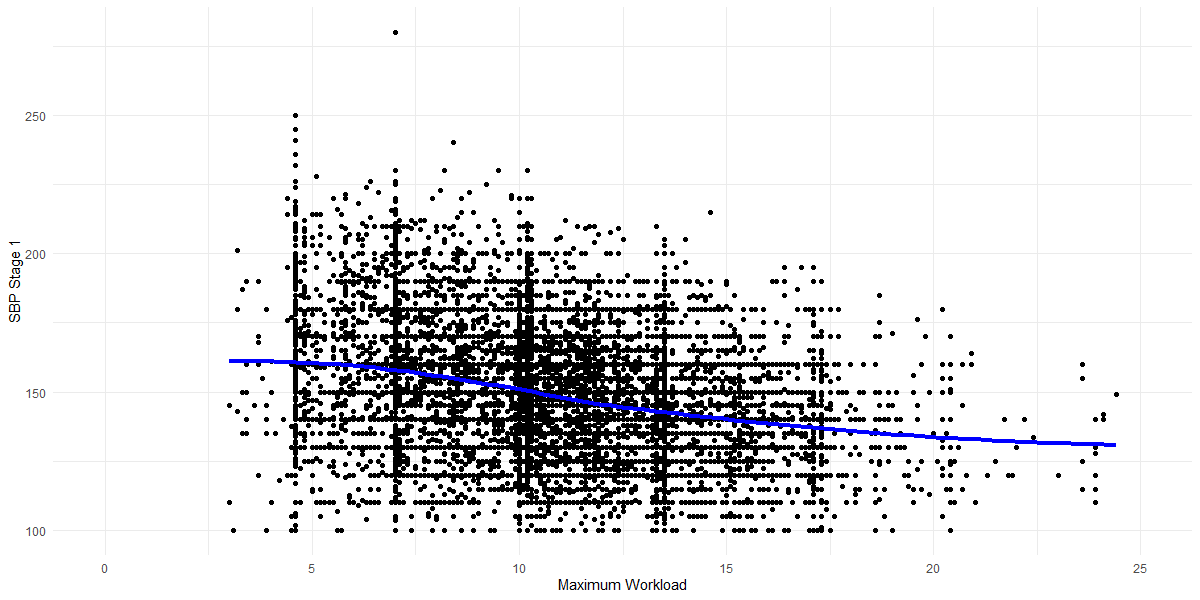
**

**Supplementary Figure 3.** Relationship between stage 1 SBP and Peak METs (maximum workload). As fitness (Peak METs) increases, the exercise SBP at stage 1 of the Bruce protocol reduces.
